# Global, regional, and national burdens of leukemia from 1990 to 2017: a systematic analysis of the global burden of disease 2017 study

**DOI:** 10.18632/aging.202809

**Published:** 2021-04-04

**Authors:** Xiangjie Lin, Jinghan Wang, Xin Huang, Huafeng Wang, Fenglin Li, Wenle Ye, Shujuan Huang, Jiajia Pan, Qing Ling, Wenwen Wei, Shihui Mao, Yu Qian, Jie Jin, Jiansong Huang

**Affiliations:** 1 Department of Hematology, The First Affiliated Hospital, Zhejiang University School of Medicine, Hangzhou, Zhejiang, China; 2 Key Laboratory of Hematologic Malignancies, Diagnosis and Treatment, Hangzhou, Zhejiang, China; 3 Institute of Hematology, Zhejiang University School of Medicine, Hangzhou, Zhejiang, China; 4 Department of Hematology, The First Affiliated Hospital of University of Science and Technology of China, Hefei, Anhui, China; 5 Zhejiang University Cancer Center, Hangzhou, Zhejiang, China

**Keywords:** leukemia, global cancer burden, prevalence, incidence, estimated annual percentage change

## Abstract

We described the spatial and temporal trends of the annual leukemia incidence, prevalence, mortality, and disability-adjusted life years (DALYs) from 1990 to 2017. Leukemia case numbers and age-standardized rates (ASRs) were extracted from the Global Burden of Disease (GBD) study 2017. The estimated annual percentage change (EAPC) in the ASR was calculated using a generalized linear model with a Gaussian distribution. The risk factors for death and DALYs due to leukemia were estimated within the comparative risk assessment framework of the GBD study. Globally, the prevalence, age-standardized prevalence rate (ASPR), and EAPC in leukemia cases in 2017 were 2.43 (95% uncertainty interval (UI) 2.19 to 2.59) million, 32.26 (95% UI 29.02 to 34.61), and 0.22% (95% CI 0.13 to 0.31, P<0.01), respectively, during 1990-2017. The trends of the age-standardized incidence, deaths, and DALY rate all significantly decreased globally. The burden of leukemia was higher in males than in female. An increasing leukemia burden was found in high-middle-sociodemographic index (SDI) countries and territories. The burden of leukemia tended to be lower in high-SDI regions than that in lower SDI regions. The rapid increases in the prevalent cases and prevalence rate of leukemia is urgent to be solved in the future.

## INTRODUCTION

Leukemia is a blood-related malignancy characterized by transformed hematopoietic progenitors and by diffuse infiltration of bone marrow. It was the 11^th^ leading cause of cancer-related mortality worldwide in 2018 [1]. Leukemia accounted for approximately 3.4% of all new cancer cases and 3.8% of all cancer deaths in 2020 according to the Surveillance, Epidemiology, and End Results (SEER) Program [2]. With the advent of new treatments, such as mutational targeted inhibitors, proapoptotic agents, chimeric antigen receptor (CAR) T-cell therapy, and immunotherapy [3, 4], mortality due to leukemia has descended recently, but leukemia is still a highly prevalent disease that leads to considerable disability and increased economic costs. It not only results in a major personal burden, but also affects families and the economic structures of countries. Recently, some studies have reported the descriptive epidemiology of leukemia based on the SEER results [5, 6], however, the burden of leukemia has not been comprehensively evaluated, despite some reviews reporting the epidemiological characteristics of some specific subtypes of leukemia [7–9]. A systematic analysis will help quantify health loss due to leukemia and guide policy-making by healthcare providers aimed at improving health systems and decreasing the burden of leukemia over time. There have been some studies on the global burden of Hodgkin lymphoma and the burden of multiple myeloma in China [10, 11], but there is still a lack of studies on leukemia in the field of hematological diseases. To provide comparable and up-to-date information on the leukemia burden, we report the global-, regional-, national-, and territorial-level incidences of, prevalences of, mortality due to, and disability-adjusted life years (DALYs) associated with leukemia and its subtypes, presenting case counts, ASRs, and trends from 1990-2017 by age, sex, and sociodemographic index (SDI).

## RESULTS

### Global level

**Figure 1:**
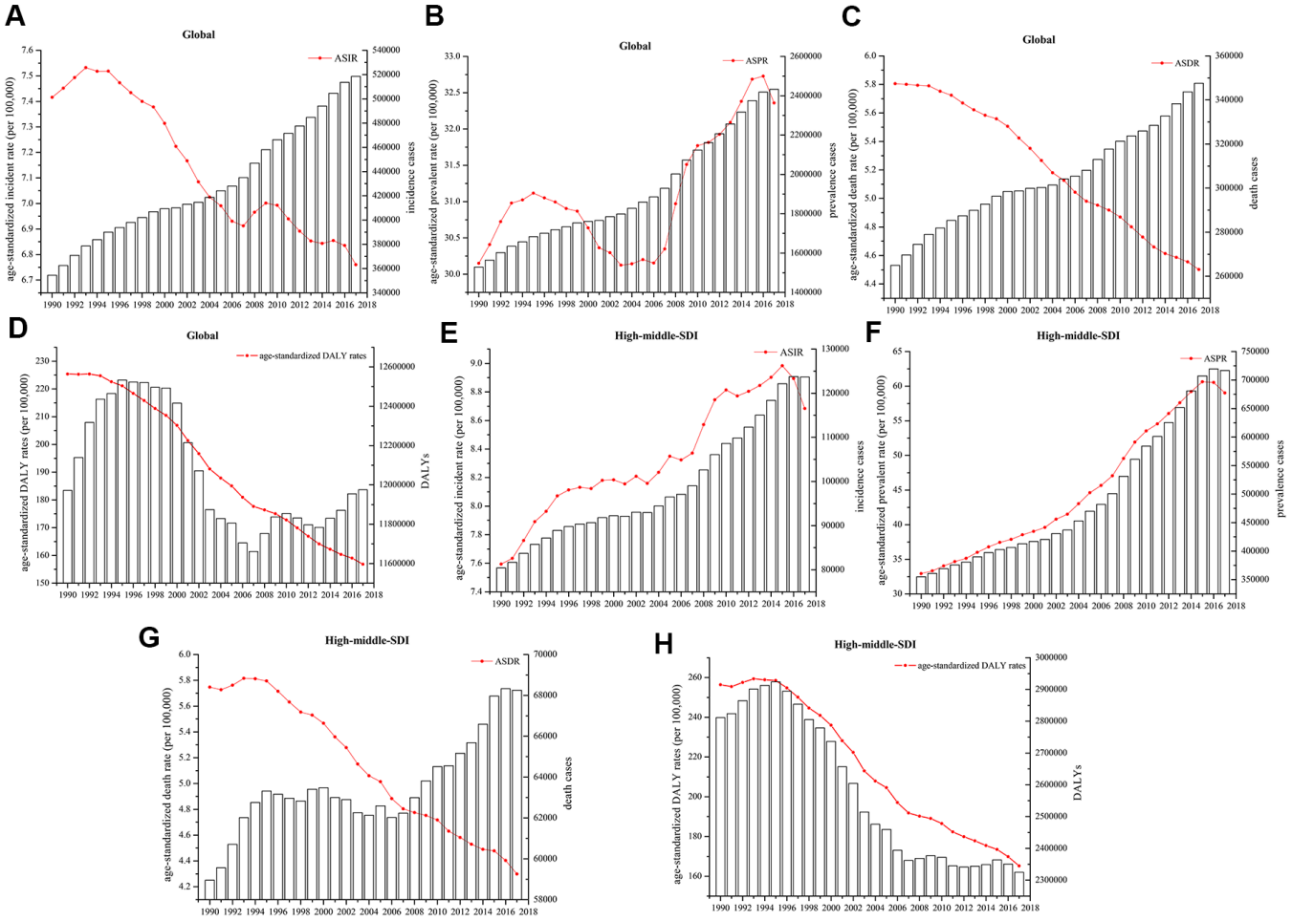
Numbers of cases and age-standardized (**A**, **E**) incidence, (**B**, **F**) prevalence, (**C**, **G**) death, and (**D**, **H**) DALY rates globally and in high-middle-SDI countries and territories.

There were approximately 2.43 million (95% UI 2.19 million to 2.59 million) prevalent cases of leukemia, with an age-standardized prevalence rate (ASPR) of 32.26 (95% UI 29.02 to 34.61) per 100,000 population in 2017. The estimated annual percentage change (EAPC) in the ASPR was 0.22% (95% CI 0.13 to 0.31, P<0.01) at the global level from 1990 to 2017. The DALYs attributable to leukemia were stable over the past 28 years, while the age-standardized DALY rate decreased significantly, with an EAPC of -1.52% (95% CI -1.60% to -1.44%, P<0.01), decreasing from 225.37 (95% UI 259.26 to 190.20) to 156.83 (95% UI 168.08 to 140.81) per 100,000 population. In addition, there were approximately 0.52 million (95% UI 0.47 million to 0.55 million) incident cases of leukemia in 2017, with an age-standardized incidence rate (ASIR) of 6.76/100,000 (95% UI 6.15 to 7.16) population and an EAPC of -0.42% (95% CI -0.48% to -0.38%, P<0.01) from 1990 and 2017. Globally, approximately 0.35 million (95% UI 0.32 million to 0.36 million) deaths were attributable to leukemia, with an age-standardized death rate (ASDR) of 4.50 (95% UI 4.12 to 4.73). The ASDR decreased by -1.04% (95%CI -1.10% to -0.99%, P<0.01) (Table 1 and Figure 1A–1D). The cases and ASRs in other regions, including low-, low-middle-, middle-, and high-SDIs regions, are showed in Supplementary Figure 1.

**Table 1:** The number cases, age-standardized rates, and EAPCs in incidence and prevalence for leukemia.

	**Incidence**					**Prevalence**			
	**Number, 2017** **(95% UI)**	**ASR/100,000,** **2017 (95% UI)**	**EAPC(%) 1990 to 2017 (95% CI)**	**P**		**Number, 2017** **(95% UI)**	**ASR/100,000,** **2017 (95% UI)**	**EAPC(%), 1990 to 2017 (95% CI)**	**P**
**Global**	518485 (472240 to 548018)	6.76 (6.15 to 7.16)	-0.42 (-0.48 to -0.38)	<0.01		2432388 (2190332 to 2591602)	32.36 (29.02 to 34.61)	0.22 (0.13 to 0.31)	<0.01
**Sex**									
Male	295386 (262628 to 314881)	8.09 (7.25 to 8.62)	-0.26 (-0.32 to -0.21)	<0.01		1337374 (1178388 to 1440142)	36.33 (32.14 to 39.18)	0.43 (0.34 to 0.53)	<0.01
Female	223099 (193548 to 239786)	5.62 (4.85 to 6.07)	-0.65 (-0.70 to -0.60)	<0.01		1095013 (941509 to 1202828)	28.75 (24.65 to 31.83)	-0.03 (-0.12 to 0.06)	0.446
**Social-demographic index**									
Low SDI	42637(36388 to 47928)	4.14(3.51 to 4.62)	-0.55(-0.60 to -0.50)	<0.01		145711(122494 to 166885)	12.68(10.66 to 14.32)	-0.82(-0.86 to -0.77)	<0.01
Low-middle SDI	66803(60348 to 76400)	4.54(4.11 to 5.21)	-0.34(-0.40 to -0.28)	<0.01		238763(212407 to 275094)	15.33(13.68 to 17.69)	-0.62(-0.68 to -0.57)	<0.01
Middle SDI	131867 (115140 to 709328)	6.47 (5.68 to 6.98)	-0.19 (-0.34 to -0.04)	0.014		644896 (561580 to 709328)	33.31 (29.06 to 37.16)	0.61 (0.33 to 0.90)	<0.01
High-middle SDI	123669 (106214 to 132373)	8.68 (7.43 to 9.46)	0.56 (0.50 to 0.63)	<0.01		716765 (599293 to 785871)	59.02 (47.60 to 66.39)	2.52 (2.37 to 2.66)	<0.01
High SDI	150825 (145068 to 156387)	7.73 (7.43 to 8.02)	-0.67 (-0.74 to -0.60)	<0.01		669515 (643865 to 693443)	44.21 (41.80 to 46.21)	0.02 (-0.09 to 0.14)	0.666
**Region**									
Central Asia	4027(3676 to 4408)	4.74(4.36 to 5.16)	-0.20(-0.31 to -0.08)	<0.01		17276 (14860 to 20638)	19.74(17.14 to 23.32)	0.04(-0.05 to 0.14)	0.371
Central Europe	11075(10405 to 11569)	6.37(5.95 to 6.72)	0.03(-0.12 to 0.17)	0.732		50380 (47177 to 53093)	35.22(31.85 to 38.95)	1.28(1.12 to 1.44)	<0.01
Eastern Europe	19717(18142 to 21322)	6.91(6.35 to 7.55)	0.06 (-0.08 to 0.21)	0.361		93861 (84126 to 104508)	37.52(31.97 to 43.78)	1.02(0.71 to 1.33)	<0.01
Australasia	4208(3602 to 4871)	9.62(8.33 to 11.02)	-1.65(-1.92 to -1.38)	<0.01		21638 (18625 to 25187)	60.56(51.70 to 70.58)	-0.96(-1.36 to -0.56)	<0.01
High-income Asia Pacific	17764(16084 to 19509)	5.27(4.80 to 5.81)	-0.47(-0.57 to -0.38)	<0.01		64160 (58413 to 70356)	30.54(26.64 to 35.38)	0.57(0.40 to 0.73)	<0.01
High-income North America	39552(38013 to 41478)	7.17(6.89 to 7.57)	-0.61(-0.78 to -0.43)	<0.01		184297(176809 to 194962)	36.74(35.07 to 38.98)	-0.41(-0.64 to -0.18)	<0.01
Southern Latin America	3932(3626 to 4260)	5.29(4.88 to 5.76)	-0.68(-0.75 to-0.61)	<0.01		14466 (12992 to 16559)	20.88(18.34 to 24.89)	-0.05(-0.12 to -0.02)	0.186
Western Europe	75694(71159 to 80241)	8.86(8.36 to 9.35)	-0.76(-0.83 to -0.69)	<0.01		323056(305276 to 341225)	49.93(46.86 to 53.40)	-0.29(-0.44 to -0.13)	<0.01
Andean Latin America	3531(2892 to 4023)	5.97(4.93 to 6.77)	0.76(-0.09 to 1.65)	0.076		13987 (11127 to 16935)	23.12(18.60 to 27.76)	-0.01(-0.16 to 0.14)	0.899
Caribbean	2797(2538 to 3200)	5.82(5.25 to 6.71)	-0.59(-0.68 to -0.50)	<0.01		10806 (9573 to 12678)	23.15(20.30 to 27.56)	-0.35(-0.44 to -0.26)	<0.01
Central Latin America	14489(13845 to 15395)	5.89(5.63 to 6.24)	-0.32(-0.38 to -0.27)	<0.01		58671 (54683 to 63720)	23.93(22.28 to 26.05)	0.10(0.06 to 0.14)	<0.01
Tropical Latin America	10170(9861 to 10602)	4.65(4.48 to 4.86)	-0.58(-0.67 to -0.49)	<0.01		34480 (32765 to 37233)	16.50(15.49 to 18.02)	-0.33(-0.41 to -0.25)	<0.01
North Africa and Middle East	32334(28685 to 36500)	6.37(5.63 to 7.19)	-0.24(-0.31 to -0.17)	<0.01		140390(124045 to 164467)	26.15(23.30 to 30.33)	0.41(0.31 to 0.50)	<0.01
South Asia	62007(53796 to 69680)	3.99(3.46 to 4.47)	-0.30(-0.39 to -0.22)	<0.01		189850(162175 to 214710)	11.71(10.02 to 13.23)	-0.68(-0.77 to -0.60)	<0.01
East Asia	147726 (124458 to 161336)	10.54 (8.81 to 11.71)	0.83 (0.63 to 1.02)	<0.01		964845 (801062 to 1067449)	81.97 (66.17 to 93.10)	2.95 (2.64 to3.26)	<0.01
Oceania	623(483 to 802)	6.34(4.99 to 7.65)	-0.07(-0.11 to -0.03)	<0.01		2156 (1610 to 2921)	20.16(15.69 to 25.41)	-0.02(-0.08 to -0.03)	0.341
Southeast Asia	38894(32374 to 42316)	3.99(3.46 to 4.47)	0.07(-0.04 to 0.19)	0.182		141696(116203 to 155338)	11.71(10.02 to 13.23)	-0.08(-0.02 to 0,02)	0.143
Central Sub-Saharan Africa	3402(2400 to 4400)	3.87(2.90 to 4.60)	-0.24(-0.27 to -0.20)	<0.01		12447 (7939 to 18702)	11.76(8.41 to 12.41)	-0.43(-0.48 to -0.38)	<0.01
Eastern Sub-Saharan Africa	13301(10190 to 16009)	4.48(3.52 to 5.21)	-0.29(-0.38 to -0.20)	<0.01		49051 (36380 to 61198)	14.17(10.82 to 16.83)	-0.31(-0.41 to -0.21)	<0.01
Southern Sub-Saharan Africa	2491(2014 to 2709)	4.10(3.35 to 4.44)	-0.31(-0.69 to -0.08)	0.117		7645 (6095 to 8688)	12.21(9.83 to 13.68)	-0.45(-0.80 to -0.08)	0.017
Western Sub-Saharan Africa	10753(8701 to 12661)	3.40(2.76 to 4.01)	-0.14(-0.23 to -0.05)	<0.01		37230 (29402 to 44826)	10.78(8.75 to 12.91)	-0.30(-0.42 to -0.17)	<0.01

### Regional level

Regionally, the highest ASIRs of leukemia per 100,000 population in 2017 were in East Asia (10.54 (95% UI 8.81 to 11.71)), Australasia (9.62 (95% UI 8.33 to 11.02)), and Western Europe (8.86 (95% UI 8.36 to 9.35)). The lowest values were in west sub-Saharan Africa (3.40 (95% UI 2.76 to 4.01)), central sub-Saharan Africa (3.87 (95% UI 2.90 to 4.60), and south Asia (3.99 (95% UI 3.46 to 4.48)). East Asia (81.97 (95% UI 66.17 to 93.10)), Australasia (60.56 (95% UI 51.70 to 70.58)), and Western Europe (49.93 (95% UI 51.70 to 70.58)) had the highest ASPRs per 100,000 population in 2017, whereas western sub-Saharan Africa (10.78 (95% UI 8.75 to 12.92)), south Asia (11.71 (95% UI 10.02 to 12.92)), and central sub-Saharan Africa (11.76 (95% UI 8,41 to 14.41)) had the lowest ASPRs. East Asia had the highest ASIR EAPC at 0.83% (95% CI 0.63% to 1.02%, P<0.01) and the highest ASPR EAPC at 2.95% (95% CI 2.64% to 3.26%, P<0.01), while Australasia had the lowest ASIR EAPC, at -1.65% (95% CI -1.92% to -1.38%, P<0.01) and the lowest ASPR EAPC, at -0.96% (95% CI -1.36% to -0.66%, P<0.01). The EAPCs in the ASIR and ASPR for most of the regions during 1990-2017 showed decreasing trends (Table 1).

Generally, the ASDRs and age-standardized DALY rates per 100,000 population in all regions decreased over the past three decades, with the largest of ASDR EAPC in East Asia (-2.20% (95% CI -2.42% to -1.97%, P<0.01)) and age-standardized DALY EAPC in East Asia (-2.85% (95% CI -3.12% to -2.56%, P<0.01)). Oceania (212.31 (95% UI 165.99 to 268.26)), Andean Latin America (202.22 (95% UI 162.76 to 228.16)), and Southeast Asia (198.24 (95% UI 168.14 to 215.67)) had the highest age-standardized DALY rates in 2017. Conversely, the age-standardized DALY rates were lowest in high-income Asia Pacific (96.33 (95% UI 90.10 to 103.09)), Western Sub-Saharan Africa (109.20 (95% UI 88.75 to 129.33)), and Southern Sub-Saharan Africa (118.02 (95% UI 88.75 to 129.33)) (Table 2).

**Table 2:** The number cases, age-standardized rates, and EAPCs in death and DALYs for leukemia.

	**Death**					**DALYs**			
	**Number, 2017** **(95% UI)**	**ASR/100,000,** **2017 (95% UI)**	**EAPC(%) 1990 to 2017 (95% CI)**	**P**		**Number, 2017** **(95% UI)**	**ASR/100,000,** **2017 (95% UI)**	**EAPC(%), 1990 to 2017 (95% CI)**	**P**
**Global**	347583 (317256 to 364877)	4.50 (4.12 to 4.73)	-1.04 (-1.10 to -0.99)	<0.01		11975348 (10749148 to 12793575)	156.83 (140.81 to 168.08)	-1.52 (-1.60 to -1.44)	<0.01
**Sex**									
Male	197270 (178442 to 209076)	5.48 (4.98 to 5.80)	-0.86 (-0.92 to -0.81)	<0.01		6823689 (6005943 to 7296349)	180.98 (159.37 to 194.15)	-1.38 (-1.46 to -1.29)	<0.01
Female	150313 (131440 to 160485)	3.69 (3.22 to 3.95)	-1.29 (-1.34 to -1.23)	<0.01		5151659 (4419005 to 5628293)	134.19 (115.03 to 147.32)	-1.70 (-1.77 to -1.63)	0.446
**Social-demographic index**									
Low SDI	34404(29275 to 38491)	3.74(3.14 to 4.16)	-0.45(-0.49 to -0.41)	<0.01		1694141(1448538 to 1917774)	139.03(118.35 to 156.17)	-0.79(-0.84 to -0.73)	<0.01
Low-middle SDI	53872(48496 to 61899)	3.95(3.58 to 4.54)	-0.31(-0.38 to -0.25)	<0.01		2441334(2196182 to 2787396)	149.86(134.94 to 171.41)	-0.72(-0.79 to -0.65)	<0.01
Middle SDI	86970(75618 to 91484)	4.15(3.62 to 4.36)	-1.12(-1.21 to -1.10)	0.014		3395955(2967991 to 3580700)	164.96(145.24 to 174.39)	-1.72(-1.85 to -1.59)	<0.01
High-middle SDI	68250(60624 to 71357)	4.30(3.81 to 4.50)	-1.21(-1.29 to -1.12)	<0.01		2324876(2030450 to 2446361)	165.15(143.72 to 174.63)	-1.87(-2.01 to -1.73)	<0.01
High SDI	102825 (100311 to 105122)	4.81 (4.69 to 4.93)	-1.16 (-1.24 to -1.08)	<0.01		2071549 (2011939 to 2129540)	127.63 (123.22 to 131.45)	-1.73 (-1.79 to -1.67)	<0.01
**Region**									
Central Asia	2950(2770 to 3134)	3.66(3.45 to 3.87)	-0.29(-0.44 to -0.14)	<0.01		133746(123327 to 143566)	151.48(140.06 to 162.45)	-0.80(-0.97 to -0.64)	<0.01
Central Europe	9758(9205 to 10099)	4.94(4.66 to 5.11)	-0.28(-0.40 to -0.15)	<0.01		225369(214138 to 234061)	141.42(133.16 to 147.76)	-1.05(-1.13 to -0.10)	<0.01
Eastern Europe	13492(13062 to 13923)	4.42(4.25 to 4.59)	-0.53(-0.70 to -0.35)	<0.01		388544(371509 to 406622)	151.71(143.72 to 160.56)	-1.39(-1.54 to -1.24)	<0.01
Australasia	2649(2414 to 2889)	5.76(5.25 to 6.26)	2.15(-2.39 to -192)	<0.01		58042(52667 to 63301)	157.93(143.40 to 171.68)	-2.30(-2.52 to -2.07)	<0.01
High-income Asia Pacific	12211(11728 to 12672)	3.13(2.98 to 3.28)	-1.37(-1.43 to -1.31)	<0.01		254074(241014 to 267087)	96.33(90.10 to 103.09)	-2.30(-2.38 to -2.22)	<0.01
High-income North America	31902(30999 to 32959)	5.38(5.22 to 5.58)	-0.99(-1.08 to -0.89)	<0.01		658363(634856 to 688336)	134.53(129.66 to 141.53)	-1.53(-1.63 to -1.43)	<0.01
Southern Latin America	3614(3338 to 3917)	4.66(4.32 to 5.03)	-0.78(-0.87 to -0.70)	<0.01		105349(97670 to 114021)	151.71(140.79 to 164.08)	-1.03(-1.09 to -0.97)	<0.01
Western Europe	48524(46559 to 50340)	5.38(5.15 to 5.59)	-0.97(-1.09 to -0.84)	<0.01		901173(857330 to 941130)	134.40(127.62 to 140.98)	-1.60(-1.74 to -1.47)	<0.01
Andean Latin America	2826(2375 to 3140)	4.91(4.14 to 5.43)	-0.04(-0.17 to -0.09)	0.554		123079(98299 to 139241)	202.22(162.76 to 228.16)	-0.30(-0.44 to -0.16)	<0.01
Caribbean	2451(2250 to 2755)	5.01(4.58 to 5.67)	-0.66(-0.74 to -0.58)	<0.01		83319(74161 to 99501)	178.65(158.01 to 215.16)	-0.87(-0.97 to -0.77)	<0.01
Central Latin America	11484(11000 to 12054)	4.75(4.55 to 4.98)	-0.53(-0.57 to -0.49)	<0.01		487962(466425 to 517540)	193.57(185.10 to 205.08)	-0.63(-0.67 to -0.58)	<0.01
Tropical Latin America	8908(8683 to 9158)	4.02(3.91 to 4.13)	-0.62(-0.70 to -0.54)	<0.01		307520(296679 to 317064)	142.02(136.27 to 146.91)	-1.00(-1.11 to -0.90)	<0.01
North Africa and Middle East	25096(22072 to 28531)	5.36(4.71 to 6.12)	-0.49(-0.55 to -0.43)	<0.01		1057846(936255 to 1198015)	190.11(168.93 to 215.27)	-0.71(-0.78 to -0.64)	<0.01
South Asia	52412(45681 to 58699)	3.57(3.11 to 3.99)	-0.21(-0.29 to -0.13)	<0.01		2248367(1946541 to 2543111)	132.77(115.04 to 149.91)	-0.62(-0.73 to -0.51)	<0.01
East Asia	63510(53813 to 68517)	3.79(3.20 to 4.09)	-2.20(-2.42 to -1.97)	<0.01		2431874(2049307 to 2608079)	171.51(144.84 to 185.33)	-2.85(-3.15 to -2.56)	<0.01
Oceania	491.40(384.82 to 613.61)	5.60(4.41 to 6.62)	-0.09(-0.13 to -0.04)	<0.01		24756(19016 to 32551)	212.31(165.99 to 268.26)	-0.09(-0.15 to -0.04)	<0.01
Southeast Asia	31823(26195 to 34581)	5.35(4.39 to 5.80)	-0.09(-0.22 to 0.04)	0.154		1264403(1070740 to 1378398)	198.24(168.14 to 215.67)	-0.39(-0.53 to -0.26)	<0.01
Central Sub-Saharan Africa	2693(1956 to 3222)	3.61(2.68 to 4.38)	-0.16(-0.18 to -0.13)	<0.01		143926(98777 to 198826)	128.16(93.27 to 152.10)	-0.37(-0.42 to -0.33)	<0.01
Eastern Sub-Saharan Africa	10133(7899 to 12012)	4.04(3.21 to 4.69)	-0.25(-0.33 to -0.18)	<0.01		560133(424475 to 679142)	150.14(117.12 to 177.47)	-0.40(-0.50 to -0.30)	<0.01
Southern Sub-Saharan Africa	2298(1892 to 2490)	4.03(3.33 to 4.36)	-0.24(-0.60 to -0.12)	0.180		80447(64346 to 88754)	118.02(95.29 to 129.37)	-0.47(-0.94 to -0.00)	0.051
Western Sub-Saharan Africa	8355(6749 to 9864)	3.11(2.52 to 3.69)	-0.07(-0.14 to -0.01)	0.038		437056(351917 to 517605)	109.20(88.75 to 129.33)	-0.31(-0.43 to -0.19)	<0.01

### National and territorial level

The ASIRs of leukemia during 1990-2017 varied across 195 countries and territories. These rates ranged from 2.56 to 14.83 per 100,000 population in 2017. Syria (14.83 (95% UI 12.15 to 17.71)), the United Kingdom (12.13 (95% UI 11.68 to 12.58)), and Denmark (11.98 (95% UI 10.41 to 13.82)) were the countries with the highest ASIRs in 2017. Malawi (2.56 (95% UI 1.94 to 3.06)), Morocco (2.74 (95% UI 2.20 to 3.29)), and Uganda (2.82 (95% UI 2.23 to 3.29)) had the lowest ASIRs of leukemia. The ASIR EAPCs from 1990 to 2017 differed substantially among countries and territories. Slovakia (1.80% (95% CI 1.59% to 2.00%, P<0.01)), Jamaica (1.52% (95% CI 1.02% to 2.02%)), and Taiwan (Province of China) (1.39% (95% CI 1.24% to 1.52%, P<0.01)) showed the largest significant increases. Bahrain (-2.60% (95% CI -3.04% to -2.16%, P<0.01)), Iraq (-1.92% (95% UI -2.23% to -1.62%, P<0.01)), and Moldova (-1.81% (95% UI -2.06% to -1.56%, P<0.01)) showed the steepest decreasing trends.

**Figure 2:**
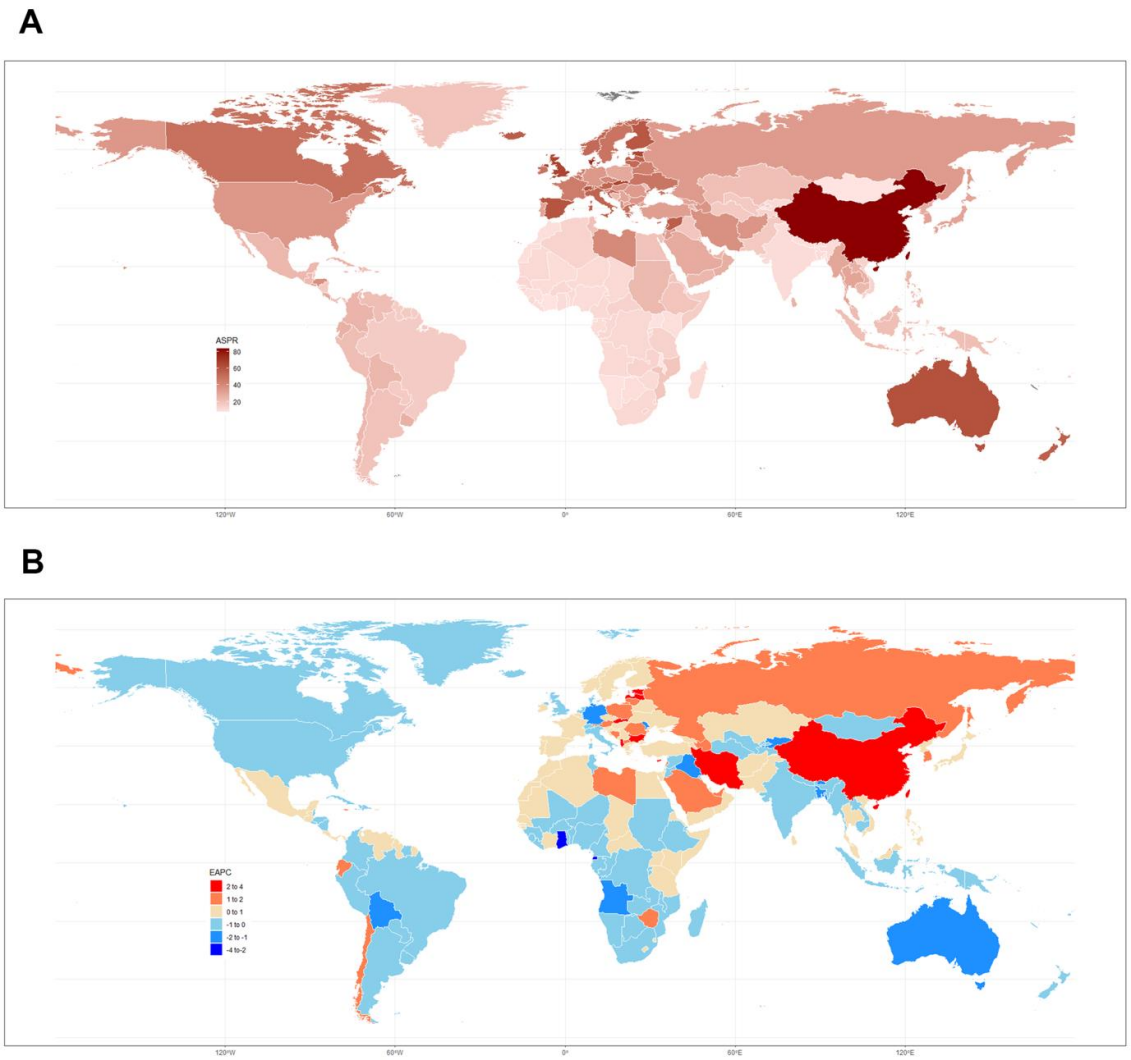
(**A**) ASPR in 2017 and (**B**) related EAPCs from 1990 to 2017 in 195 countries and territories.

The ASPR of leukemia in 2017 varied from 7.98 to 83.51 cases per 100,000 population. China (83.51 (95% UI 67.27 to 95.01)), Denmark (74.53 (95% UI 63.06 to 86.57)), and Slovakia (66.52 (95% UI 50.70 to 81.89)) had the highest ASPRs in 2017. Mongolia (7.98 (95%UI 6.22 to 10.30)), Malawi (8.08 (95%UI 5.79 to 10.08)), and Cote d’Ivoire (8.41 (95%UI 6.48 to 10.30)) had the lowest rates. Slovakia (3.60% (95% CI 3.39% to 3.81%, P<0.01)), Taiwan (Province of China) (3.00% (95% CI 2.75% to 3.25%, P<0.01)), and China (2.97% (95% CI 2.66% to 3.28%, P<0.01)) had the greatest increase from 1990 to 2017. Ghana (-2.14% (95% CI -1.69% to -2.59%, P<0.01)), Equatorial Guinea (-2.09% (95% CI -1.80% to -2.38%, P<0.01)), and Kyrgyzstan (-1.99% (95% CI -1.83% to -2.15%, P<0.01)) had the greatest decreases. The ASPRs in 2017 and the related EAPCs among 195 countries and territories are presented in Figure 2.

The countries with the highest ASDRs of leukemia in 2017 also had the highest age-standardized DALY rates, as did the countries with the lowest ASDRs. Syria, Honduras, and Afghanistan had the highest ASDRs per 100,000 population at 13.72 (95% UI 10.56 to 16.52), 9.82 (95% UI 7.96 to 12.06), and 9.74 (95% UI 7.29 to 13.16), respectively. The largest decreases in ASDRs during the 1990-2017 period were observed in Bahrain (-3.00% (95% CI -3.49% to -2.53%, P<0.01)), Finland (-2.73% (95% CI -2.98% to -2.48%, P<0.01)), Australia (-2.35% (95% CI -2.61% to -2.08%, P<0.01)), while Bahrain (-2.99% (95% CI -3.42% to -2.55%, P<0.01)), China (-2.93% (95% CI -3.23% to -2.63%, P<0.01)), and South Korea (-2.78% (95% CI -2.90% to -2.67%, P<0.01)) also showed the largest decreases in age-standardized DALY rates over the past 28 years. The details of the EAPCs for the other 195 countries are listed in Supplementary Table 4 and Supplementary Figures 2, 3.

Similar to the trends of prevalent cases and ASPRs worldwide, high-middle-SDI countries and territories had consistently increasing numbers of prevalent cases and ASPRs, and the values in 2017 were almost double those in 1990. The EAPC in the ASPR in high-middle-SDI countries and territories was 2.52% (95% CI 2.37% to 2.66%, P<0.01). The number of incident cases and the ASIR in high-middle-SDI countries and territories increased from 80,397 (95% UI 67,571 to 87,985) and 7.59 (95% UI 6.44 to 8.27) to 123,669 (95% UI 106,234 to 132,373) and 8.68 (95% UI 7.43 to 9.46), respectively. The number of deaths in high-middle-SDI countries and territories increased slightly over the past 28 years, in contrast, the ASDR decreased slightly, with an EAPC of -1.21% (95% CI -1.29% to -1.12%, P<0.01). Both the DALY and age-standardized DALY rates in the high-middle-SDI countries decreased sharply from 1990 to 2017 and with an the EAPC of -1.72% (95% CI -1.85% to -1.59%, P<0.01) (Table 1 and Figure 1E–1H).

### Sex patterns

**Figure 3:**
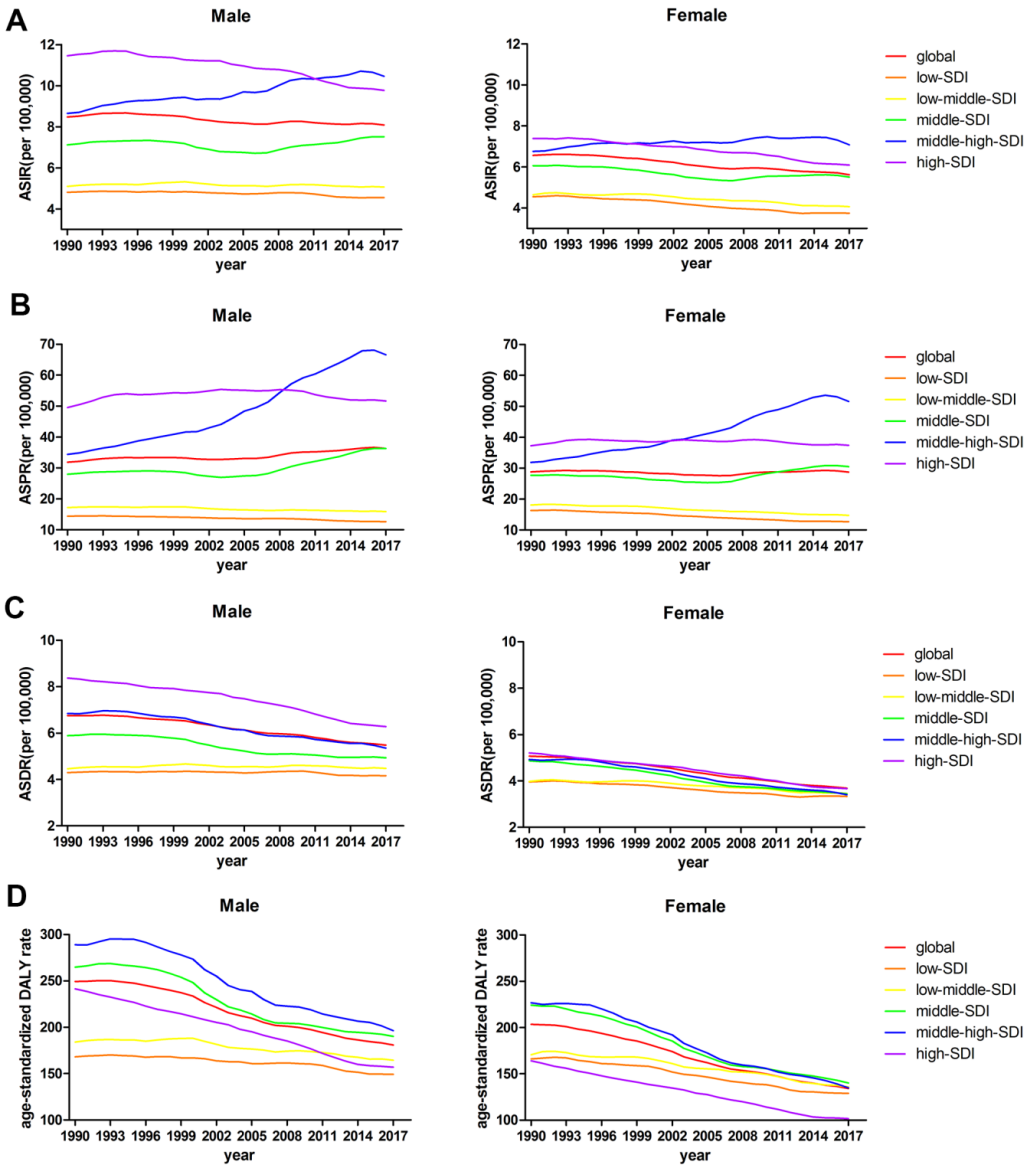
(**A**) ASIRs, (**B**) ASPRs, (**C**) ASDRs, and (**D**) age-standardized DALY rates from 1990 to 2017 by sex.

**Figure 4:**
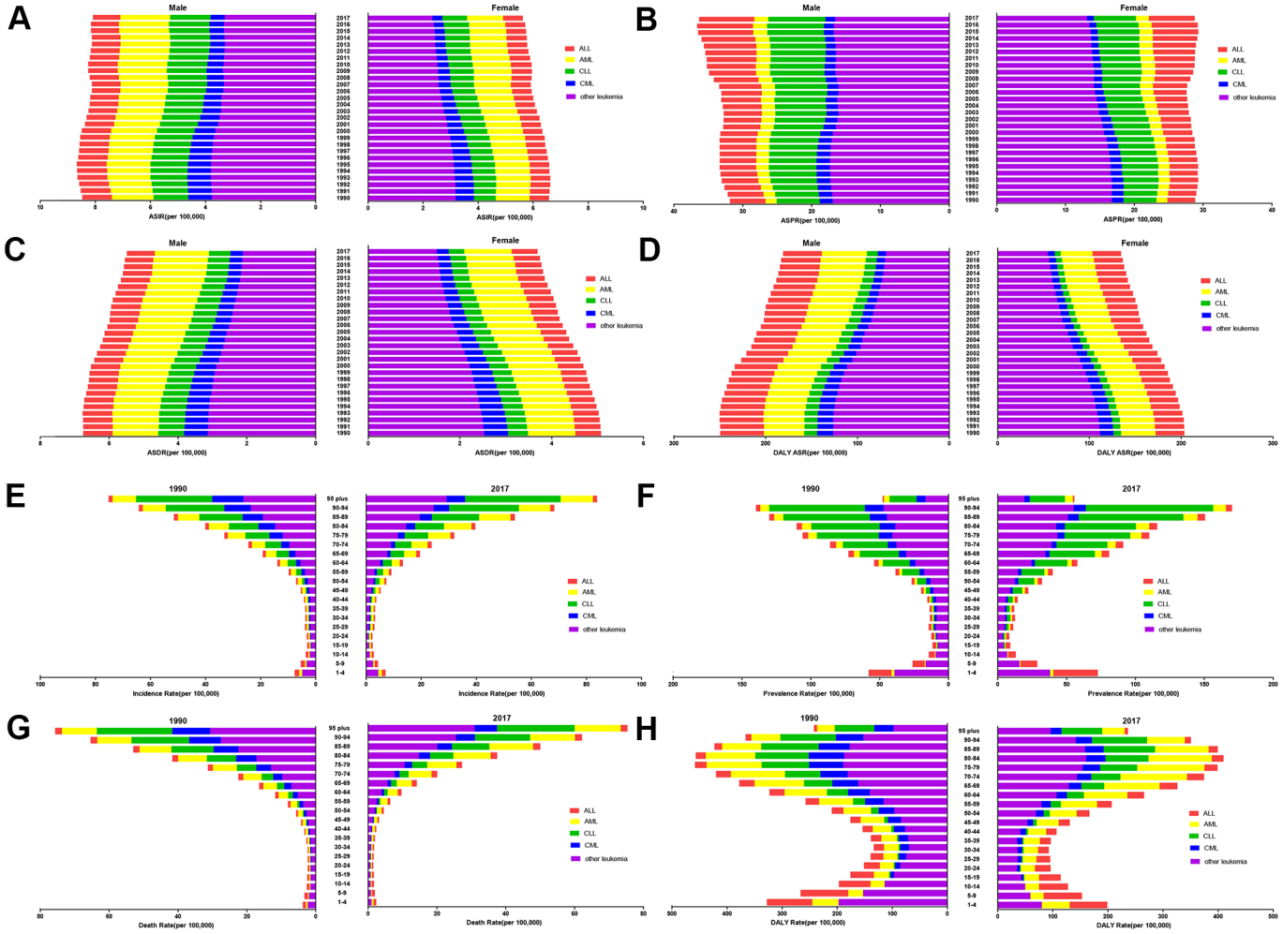
The distribution of leukemia (**A**) ASIRs, (**B**) ASPRs, (**C**) ASDRs, and (**D**) age-standardized DALY rates from 1990 to 2017 by sex. The proportions of the five subtypes leukemia in the different age groups in 1990 and 2017, and the burdens associated with the (**E**) ASIR, (**F**) ASPR, (**G**) ASDR, and (**H**) age-standardized DALY rate.

In the current study, the ASPRs of leukemia per 100,000 population were 36.33 (95% UI 32.14 to 39.18) in males and 28.75 (95% UI 24.65 to 31.83) in females in 2017. There was a moderate decline in the ASPR in females, whereas the rate was significantly increased in males during the same period. At the global level, the ASRs in high-SDI, and high-middle-SDI countries and territories were mostly higher in males than in females. In addition, there were no obvious differences in the ASRs between male and female in middle-SDI, low-middle-SDI, and low-SDI countries and territories (Figure 3). The ASPR trend increased yearly in middle-high-SDI countries and territories, peaking in 2016 in both males and females (Figure 3B). Among the ASRs observed in the past 28 years, only the ASPR in males had an increased EAPC (0.43% (95% CI 0.34% to 0.53%, P<0.01)) (Table 1). Statistics were calculated on the basis of the five types of leukemia in males and females. Among the leukemia types, acute myeloid leukemia (AML) was the leading contributor to the ASIR, ASDR, and the age-standardized DALY rate. Regarding the ASPR of leukemia in 2017, chronic lymphocytic leukemia (CLL) (8.28 (95% UI 7.66 to 8.95)) was the second leading contributor in males, whereas acute lymphocytic leukemia (ALL) (6.65 (95%UI 5.17 to 7.90)) was the second leading cause in female. chronic myeloid leukemia (CML) accounted for the smallest proportion of all ASRs (Figure 4A–4D).

### Age pattern

The trend and distribution of leukemia in 2017 was generally similar to that in 1990 by age group. The total incidence rates and prevalence rates in 2017 were slightly higher than those in 1990, while mortality and DALY rates remained stable between 1990 and 2017. The 95 and older age group had the highest incidence rate and mortality rate per 100,000 population both in 1990 and 2017, and both rates increased with age. For the incidence rate in the 95 and older age group, CLL (34.73 (95% UI 31.51 to 38.17)) accounted for the largest proportion in 2017. For the mortality rate in the 95 and older age group, other leukemia types (30.87 (95% UI 29.46 to 32.49)) accounted for the largest proportion. ALL accounted for the smallest proportion of both rates in the 95 and older age group, with an incidence rate of 1.50 (95% UI 1.42 to 1.58) and a mortality rate of 1.98 (95% UI 1.87 to 2.03) in 2017. The prevalence rate of ALL was the highest in infants and young children in 2017, especially in the group aged 1-4 years, with a prevalence rate of 32.31 (95% UI 25.14 to 38.82) per 100,000 population. CLL was the leading cause of leukemia in elderly people in both 1990 and 2017. The peak DALY rate of leukemia in 2017 occurred in the group aged 80-84 (409.53 (95% UI 384.16 to 429.09)) per 100,000 population, AML had a DALY rate of 115.08 (95% UI 106.36 to 120.29), ranking second to only other leukemia types (160.17 (95% UI 147.68 to 172.93)) (Figure 4E–4H).

### Correlation between the burden of leukemia and the sociodemographic index

**Figure 5:**
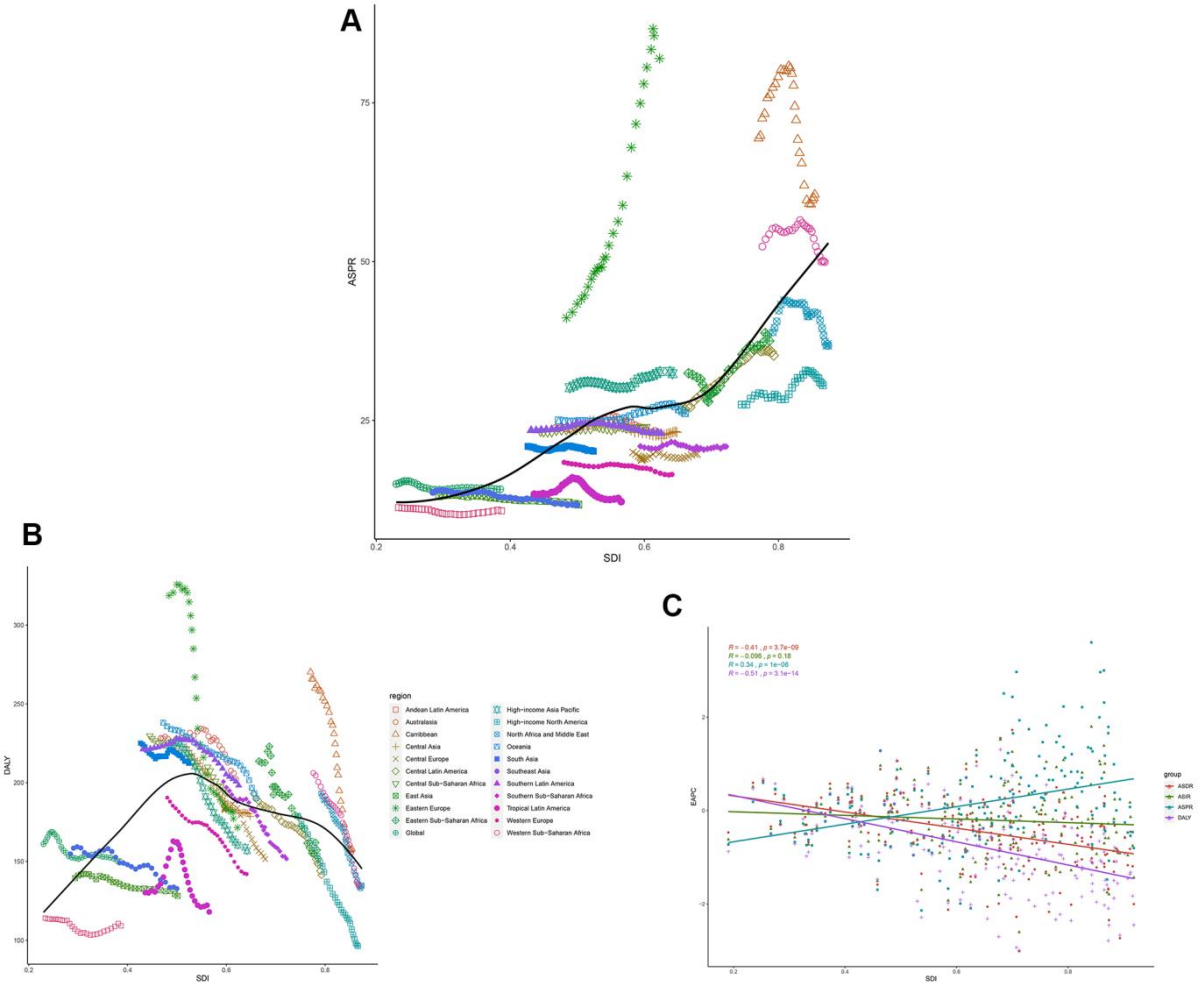
The black line represents the average expected relationship between leukemia (**A**) ASPRs and (**B**) DALYs and SDIs in GBD super regions based on values from 1990 to 2017. (**C**) The correlation of EAPCs and leukemia ASRs in 195 countries and territories in 2017.

Overall, a positive association was observed between the ASIR and SDI (Supplementary Figure 4A), as well as the ASPR and SDI (Figure 5A) for all Global Burden of Disease (GBD) regions from 1990 to 2017. At the global level, the ASIR estimates of leukemia were higher than expected in Australasia, East Asia, North Africa and the Middle East, Oceania, Southeast Asia, and Western Europe based on the SDI during 1990-2017. Moreover, globally, the ASPR estimates in only Andean Latin America, Australasia, and East Asia, were higher than expected. The ASDRs of the GBD regions increased slightly with increasing SDIs (Supplementary Figure 4B). A significant positive association between the age-standardized YLD due to leukemia and the SDI (Supplementary Figure 4C) was observed, and the middle-SDI GBD regions had the highest age-standardized DALY rates per 100,000 population (Figure 5B).

**Figure 6:**
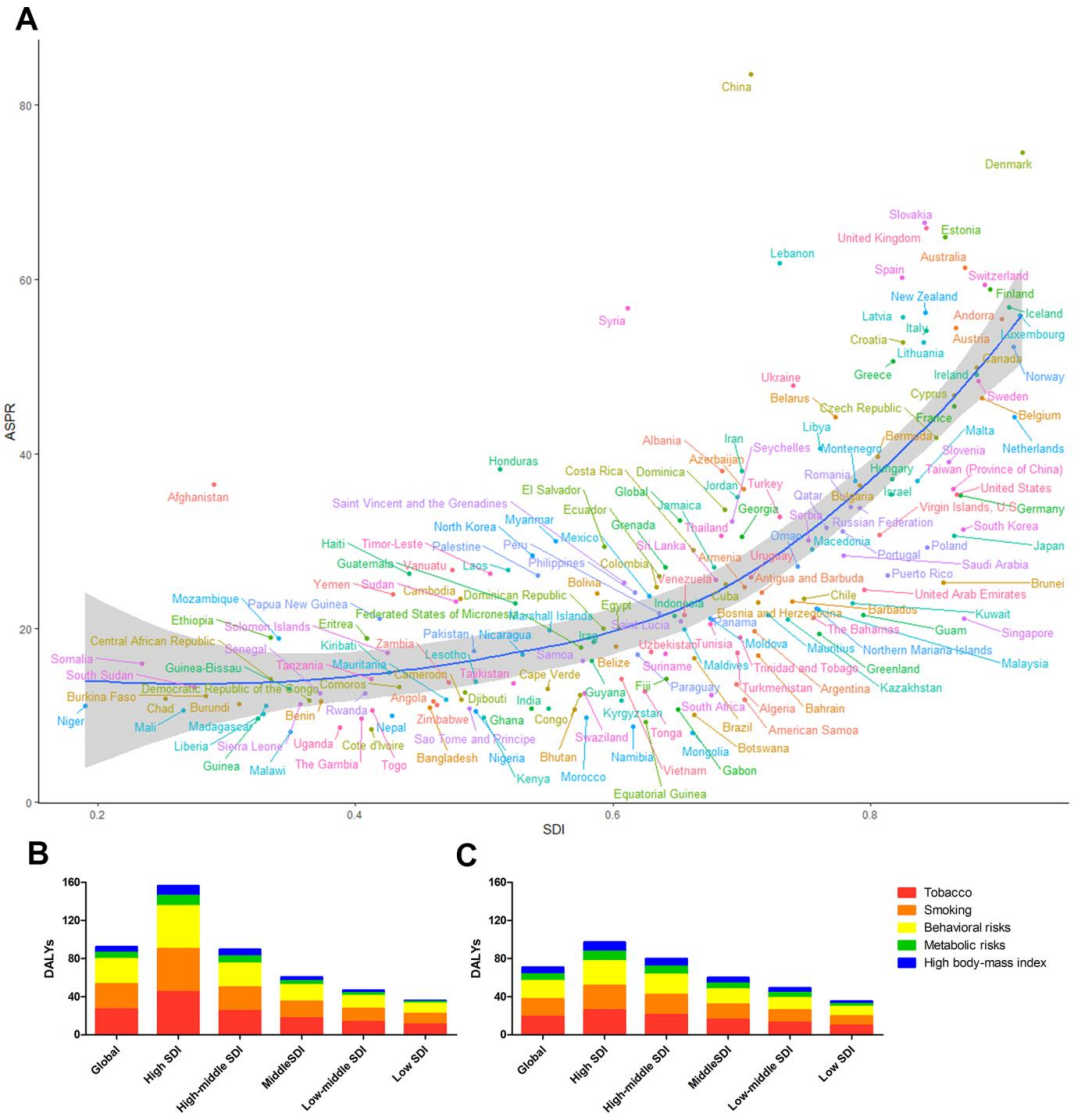
(**A**) The blue line represents the expected ASPRs and SDIs in 195 countries and territories. Each point shows the observed ASPR for a specified country in 2017. The leukemia DALYs attributable to risk factors in (**B**) 1990 and (**C**) 2017.

The same correlation was observed in the correlation analysis of the burden of leukemia in 2017 and the SDIs of 195 GBD countries and territories (Figure 6A and Supplementary Figure 5). The high burden of leukemia was not limited to developing countries such as China, Syria, Afghanistan, and Honduras. Developed countries, such as–the United Kingdom, Denmark, Slovakia, and Switzerland–had a much higher leukemia burden than expected.

We also analyzed the EAPCs of 195 countries and territories by SDI in 2017. We observed a statistically significant correlation between the EAPC in the ASPR and the SDI (R=0.34, P<0.01). In addition, a negative correlation between the change in the ASDR and SDI (R=-0.41, P<0.01), was found; this correlation was also observed for the DALYs trend (R=-0.51, P<0.01) in 2017. There was no correlation between the EAPC in the ASIR and SDI (Figure 5C).

### Risk factors contributing to the leukemia burden

Tobacco use, smoking, and behavioral risks factors were the three largest contributors to DALYs globally, especially in high-SDI countries and territories in 1990 (Figure 6B). Leukemia-related DALYs attributable to tobacco use and smoking varied globally by sex in 2017 (Figure 6C). In 1990, 34.34% (95% UI 24.02% to 44.08%) and 11.97% (95% UI 8.80% to 17.01%) of leukemia cases were attributable to tobacco use and smoking in males and females, respectively, but the leading risk factor for leukemia in females changed in 2017. Tobacco use and smoking accounted for 35.41% (95% UI 24.26% to 47.38%) of global leukemia DALYs in males in 2017, while the proportion decreased to 10.22% (95% UI 3.07% to 13.97%) in females. Metabolic risks factors and high body mass index contributed to the increase in leukemia in females in 2017 (11.55% (95% UI 5.34% to 18.97%)), overtaking tobacco use- and smoking-attributable leukemia rates in females (Supplementary Figure 6A–6D). Similarly, deaths attributable to risk factors including tobacco use, smoking, behavioral risk factors, metabolic risk factors and high body mass index had the same DALYs and risk factors patterns (Supplementary Figure 6E, 6F).

## DISCUSSION

The global and national burdens of leukemia including the incidence, prevalence, mortality, and DALYs in 195 countries and territories from 1990 to 2017 were described in this report. In 1990, there were approximately 0.35 million incident cases of, 1.53 million prevalent cases of, 0.26 million deaths due to, and 0.16 million YLDs due to leukemia, while there were 0.52 million incident cases, 2.43 million prevalent cases, 0.35 million deaths, and 0.26 million YLDs in 2017. The numbers of incident cases, prevalent cases, and YLDs increased greatly, although the ASRs of leukemia changed only slightly over the study period. The changes in the numbers of incident and prevalent leukemia cases, especially prevalent cases, were probably due to population growth and aging.

It is clear that ALL was the leading cause of prevalent cases in infants and young children, and CLL was the leading cause in elderly people, according to the prevalence rate per 1,000.000 population in 2017. The age-related mortality rates indicated that leukemia resulted in deaths most often in older patients and unhealthy young people. A previous study reported that ALL was the most commonly type of leukemia in children between the ages of 2-6 and was slightly more frequent in boys than in girls [12, 13]. The overall cure rate of childhood ALL is currently approximately 75%-80%, while for AML the cure rate is 40%-45% [14]. Environmental risk factors for childhood leukemia include ionizing and nonionizing radiation [15], chemicals, such as hydrocarbons and pesticides, and parental tobacco use [16]. Differences in prevalence and incidence between males and females were most likely due to exposure to risk factors, such as smoking and obesity. For over a decade, cigarettes have been regarded as an established risk factor for leukemia [17, 18].

Although the burden of leukemia declined significantly over time in regions with high SDIs, the data showed that these regions still had high ASRs, except for age-standardized DALY rates. The peak age-standardized DALY rates occurred in middle- and high-middle-SDI regions in 2017. Moreover, the high-middle-SDI regions had a positive EAPC in the ASIR and a considerably high EAPC in the ASPR. In recent years, second-generation tyrosine kinase inhibitors and the improvements in treatment schedules have prolonged overall survival and decreased mortality in leukemia patients, further influencing this trend [19, 20].

The GBD study 2017 provided high-quality estimates of global cancer burdens, yet there were several limitations. One is the case definitions of the various types of leukemia. Local registration and statistical systems, which are key sources of mortality data, did not cover the entire population. In these situations, autopsy data is the best source for estimating mortality rates in countries, but autopsies cannot always accurately determine cause of death. Some studies have proposed the concept of molecular epidemiology, as specific genetic mutations are key characteristics of leukemia [21, 22]. Another limitation is racial differences. We reported the estimates of leukemia burden by age and sex, but not by racial group. Leukemia occurs in all racial groups and some studies have suggested that different racial groups are affected differently by subtypes of leukemia. For example, Christopher et al [23] showed that Blacks persons with CLL had unmutated IGHV genes, chromosome 17p or 11q deletions, and increased ZAP70 expression, which may contribute to a worse prognosis, more frequently than that other races.

Country-specific information about the burden of leukemia must be provided to meet the needs of policymakers. Yi M et al [24] recently reported the burden of AML. This data can help policymakers evaluate their cancer control programs and allocate limited medical resources in health care systems. Given that most existing data are not of high quality, the results of the GBD study 2017 can be used to study the trends of leukemia in respective countries and territories.

In general, the global burden of leukemia increased slightly from 1990 to 2017. The increasing leukemia burden was mainly observed in high-middle SDI countries and territories, signifying the need for greater investments in research to reduce the incidence and prevalence of leukemia in developing countries and territories. Moreover, high-middle-SDI countries and territories should focus on improving leukemia treatment. The burden of leukemia tended to be low in high SDI-regions, though these regions still a higher burden than lower SDI-regions. The prevalence showed a bimodal distribution and was increased in people aged 1-4 and 90-94 years, highlighting the need to improve leukemia screening in these two age groups. A higher burden of leukemia was observed in males than in females, and this difference has to be taken into account by policymakers when adjusting prevention strategies. Of the five subtypes of leukemia, other leukemia types require additional attention in the future. The rapid increase in the number of prevalent cases and the prevalence rate of leukemia are problems that need to be considered.

## MATERIALS AND METHODS

### Data source

Data on the incidence, prevalence, mortality, DALYs, and years lived with disability (YLDs) of leukemia from 1990 to 2017 were obtained from the Global Health Data Exchange (GHDx) query tool (http://ghdx.healthdata.org/gbd-results-tool), which is a catalog of global health and demographic data from the GBD study 2017. The GBD study 2017, conducted by the Institute of Health Metrics and Evaluation (IHME), is the largest and most complete study to date, providing data on epidemiological characteristics and worldwide trends. Twenty-one regions, 7 super-regions and 195 countries and territories were included in the GBD study 2017.

### Case definition

The subtypes of leukemia–ALL, AML, CLL, and CML–were identified based on the International Classification of Diseases and Injuries-10^th^ edition (ICD-10) diagnostic codes (Supplementary Table 1). The current study employed the GBD study 2017 definition of the prevalence of leukemia: a diagnosis and primary therapy phase of 5 months, a metastatic phase of 43.67 months, and a terminal phase of 1 month. All the phases were presented with their respective disability weights (Supplementary Table 2) [25, 26]. In addition, survivals beyond ten years was considered cured. A systematic review of the incidence and prevalence of leukemia was performed for 1980-2015 for the GBD study 2016 and updated for the GBD study 2017 [26]. The standardized methods of the GBD study 2017 have been reported elsewhere [27]. The prevalence and DALYs of leukemia were estimated for 195 countries and territories using DisMod-MR 2.1, a Bayesian meta-regression tool [26]. All estimates are presented as counts, computed from the means of 1000 draws, and 95% uncertainty intervals (95% UI) were determined using 97.5% and 2.5% of the distribution of draws. To compute YLDs for a particular health outcome in a given population, the number of people living with that outcome is multiplied by a disability weight that represents the magnitude of health loss associated with the outcome of interest [27]. Years of life lost (YLLs) due to leukemia were calculated using standard global life expectancy values and the number of deaths according to age [27]. Leukemia DALYs were computed as the sum of YLDs and YLLs.

### Sociodemographic index

The SDI is a composite indicator of development status and was obtained from the GBD study. It was calculated from a geometric mean ranging from 0 to 1, which accounts for average educational attainment in the population older than 15 years (EDU15+), total fertility rate in those under 25 years (TFU25), and lag-distributed income (LDI) per capita [28], with SDI scales to confirm the minima and maxima, which are listed in Supplementary Table 3. The index scores for calculating the SDIs were computed as follows: I_Cly_= (C_ly_- C_low_)/(C_high_- C_low_), where I_Cly_ is the index value for covariate C, location l, and year y. In addition, the index value for TFU25 was calculated as (1-I_TFU25ly_); a lower TFU25 corresponds to a higher level of development. Countries and territories were classified as regions with low, low-middle, middle, high-middle, and high SDIs.

### Statistical analyses

The ASR per 100,000 population is equal to the sum of the product of the specific age ratio (*a*_i_) in age group *i* and the number or weight (*w*_i_) of the selected reference standard population group *i* divided by the sum of the number or weight of the standard population. ASRs were calculated on the basis of the following formula:

$$\text{ASR}=\frac{\sum_{i=1}^{A}aiwi}{\sum_{i=1}^{A}wi}\times 100,000$$

The EAPC was used to quantify the trends of the ASRs over a specified interval, which were computed using a generalized linear model with a Gaussian distribution [29–31]. A regression line was fitted to the natural logarithm of the rates, i.e., y =α+βx+ε, where x = year and y = ln(rate), from which 95% confidence intervals were obtained; the EAPC was calculated as 100 × (e^β^ - 1). A positive EAPC indicated an upward ASR trend, whereas a negative EAPC indicated a downward trend. Spearman’s rank order correlation was used to measure the associations between ASRs and SDIs, and the Pearson correlation coefficient was used to quantify the correlation between EAPCs and SDI from 1990 to 2017. A P value of less than 0.05 was regarded as statistically significant. All statistical analyses were performed using the R program (Version 3.6.3).

### Availability of data and materials

All data and materials supporting the conclusions of this study have been included within the article.

## Supplementary Material

Supplementary Figures

Supplementary Tables 1, 2 and 3

Supplementary Table 4
