# Supplementary material for: Global, regional, and national burdens of leukemia from 1990 to 2017: a systematic analysis of the global burden of disease 2017 study
Source: Aging (Albany NY). 2021 Apr 4;13(7):10468–89. doi: 10.18632/aging.202809 (PMC8064161; doi:10.18632/aging.202809)
Supplement: Supplementary Figures [file aging-13-202809-s001.pdf]

SUPPLEMENTARY FIGURES

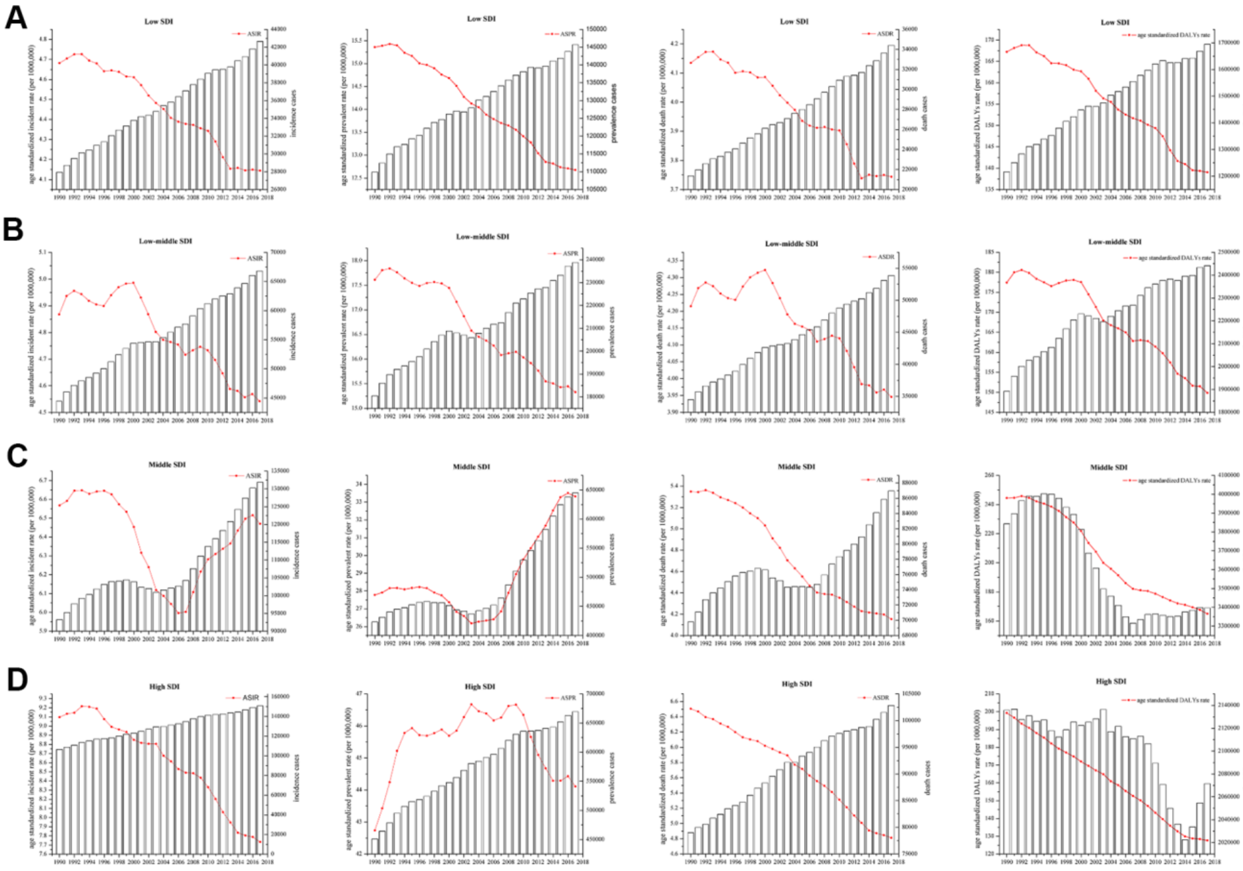

Supplementary Figure 1. Numbers of cases and age-standardized incidence, prevalence, death, and DALY rates in (**A**) low-, (**B**) low-middle-, (**C**) middle-, and (**D**) high-SDI countries and territories.

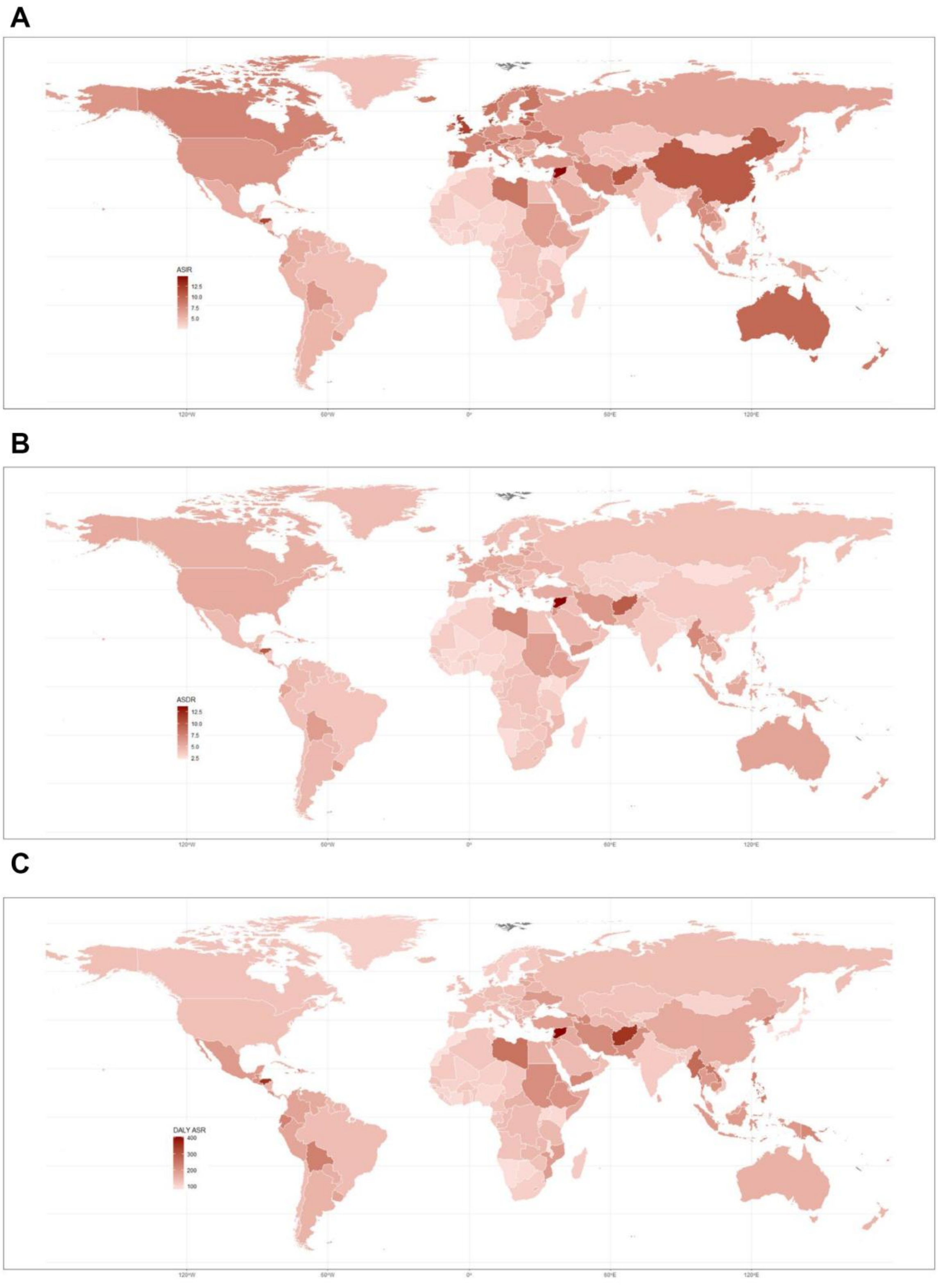

Supplementary Figure 2. Age-standardized (**A**) incidence, (**B**) deaths, and (**C**) DALYs in 195 countries and territories in 2017.

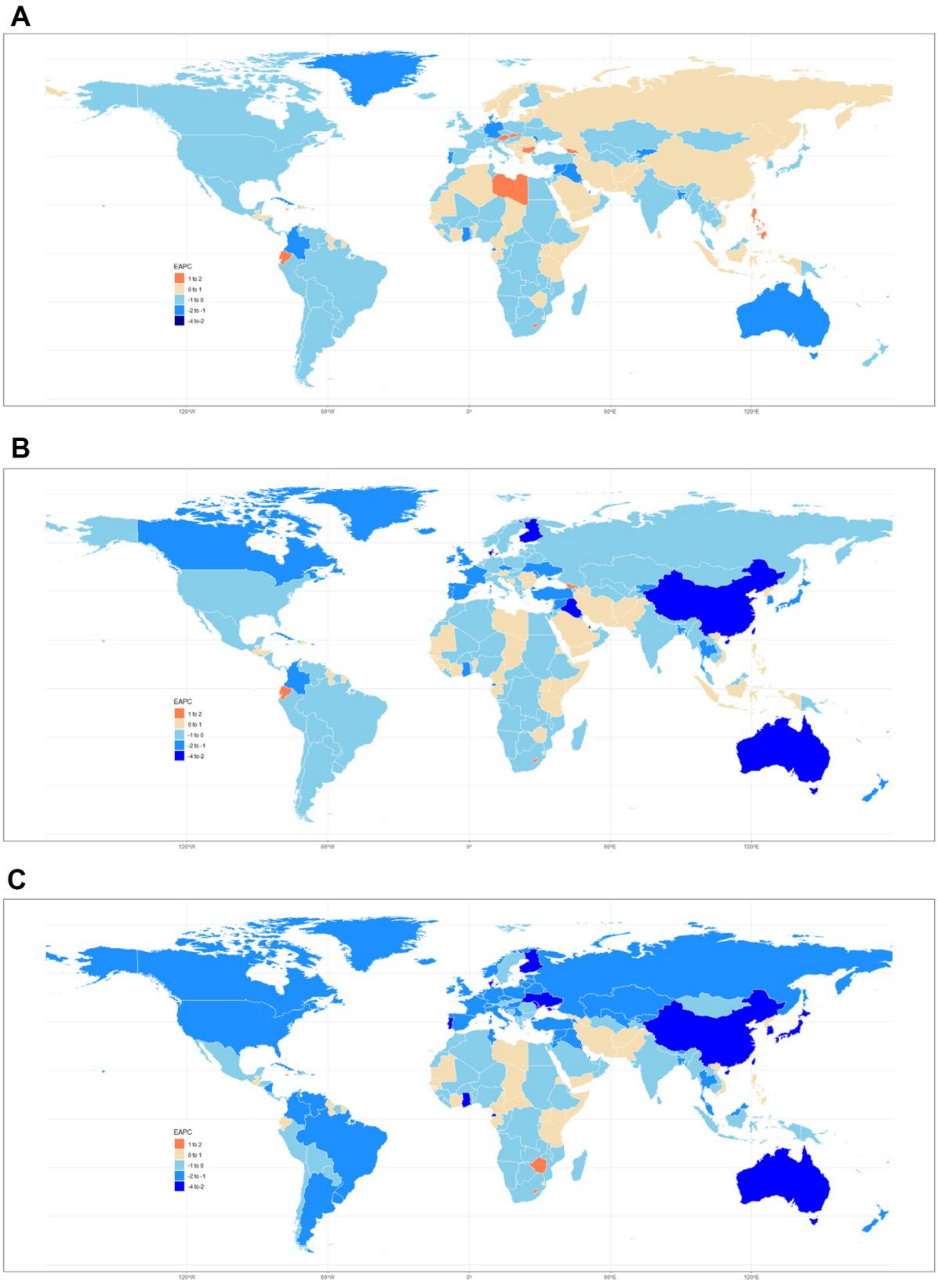

Supplementary Figure 3. EAPCs in (**A**) incidence, (**B**) deaths, and (**C**) DALYs in 195 countries and territories from 1990 to 2017.

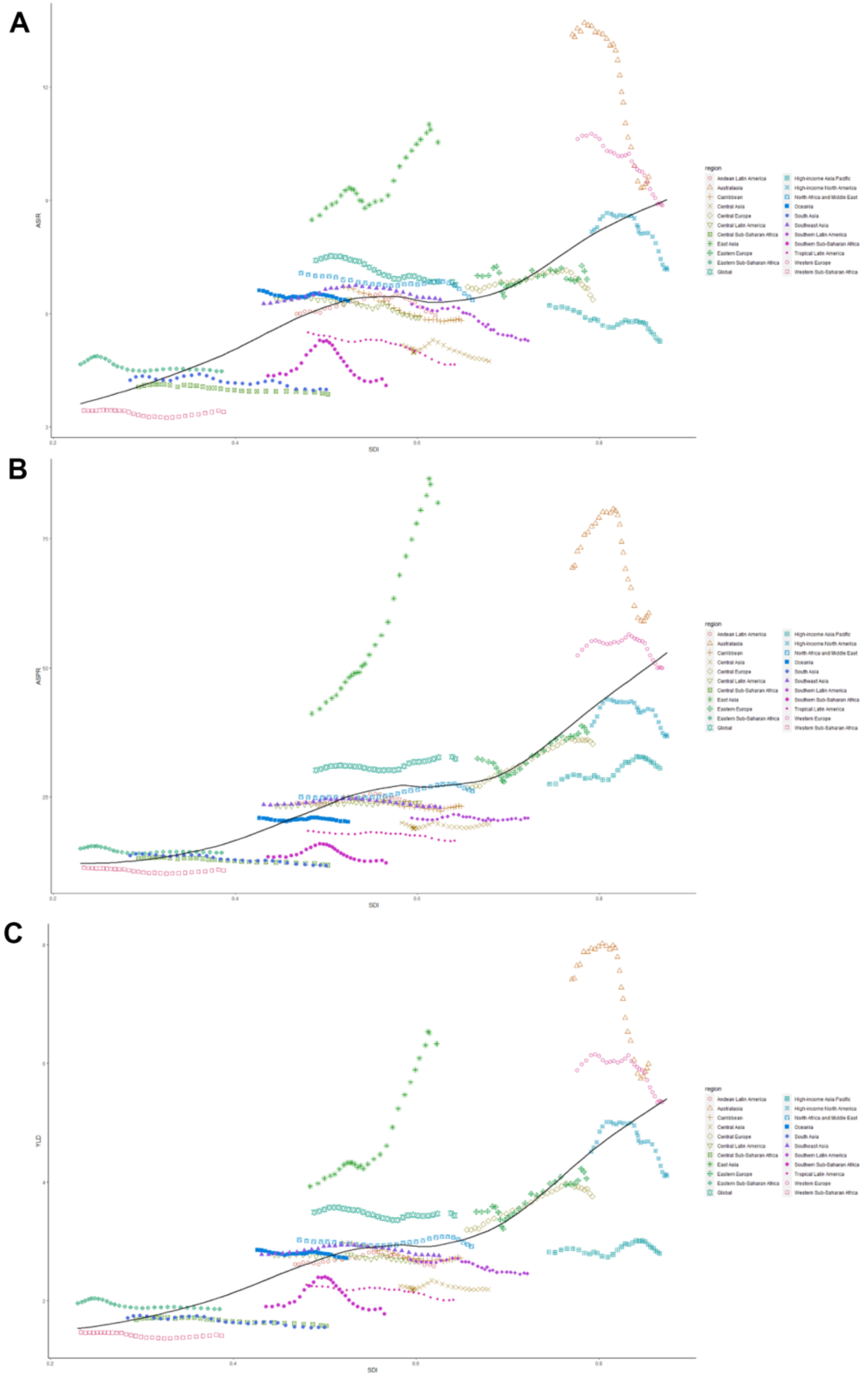

Supplementary Figure 4. The correlation between SDIs and (**A**) ASIRs, (**B**) ASDRs, and (**C**) age-standardized YLD rates.

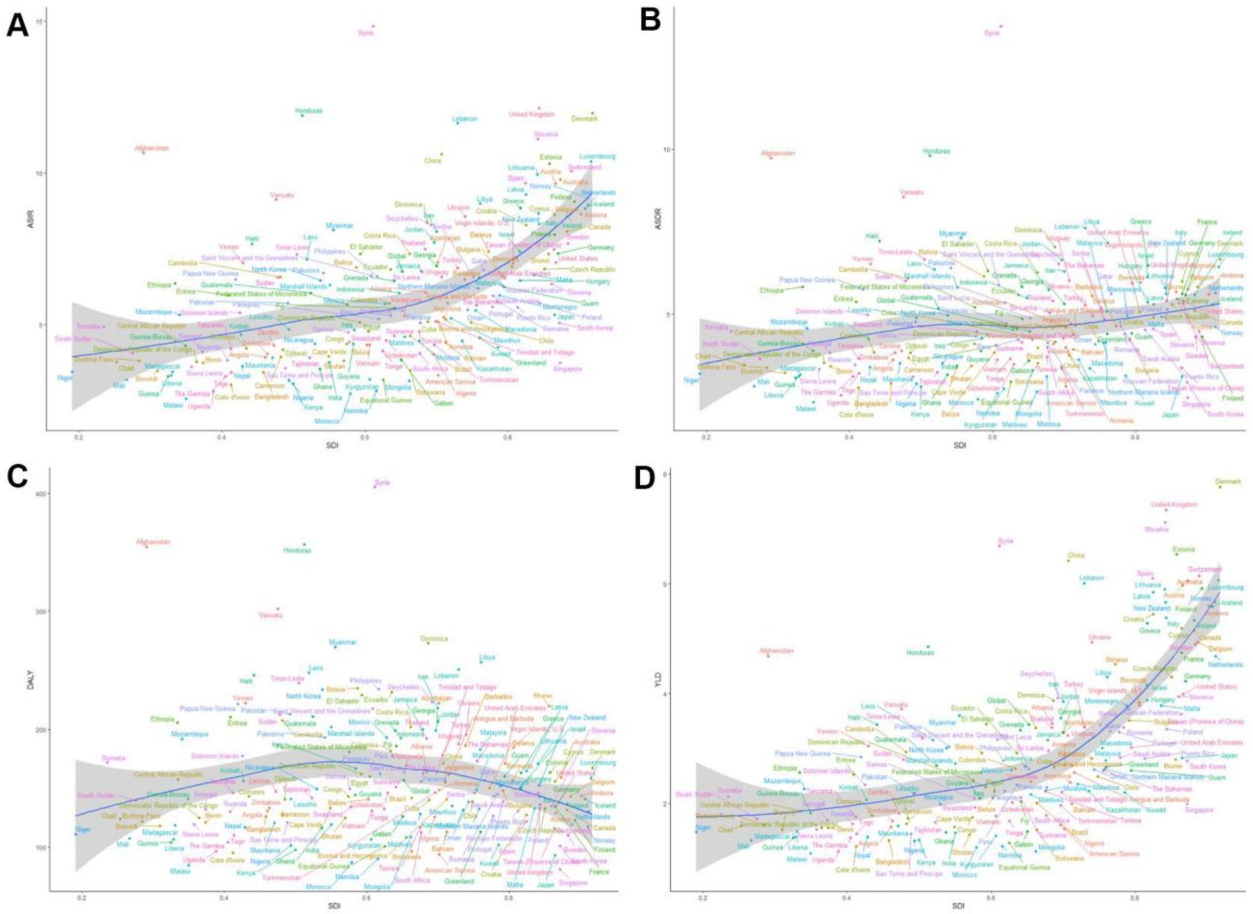

Supplementary Figure 5. The correlation between SDIs and (**A**) ASIRs, (**B**) ASDRs, (**C**) age-standardized DALY rates, and (**D**) age-standardized YLD rates in 195 countries and territories.

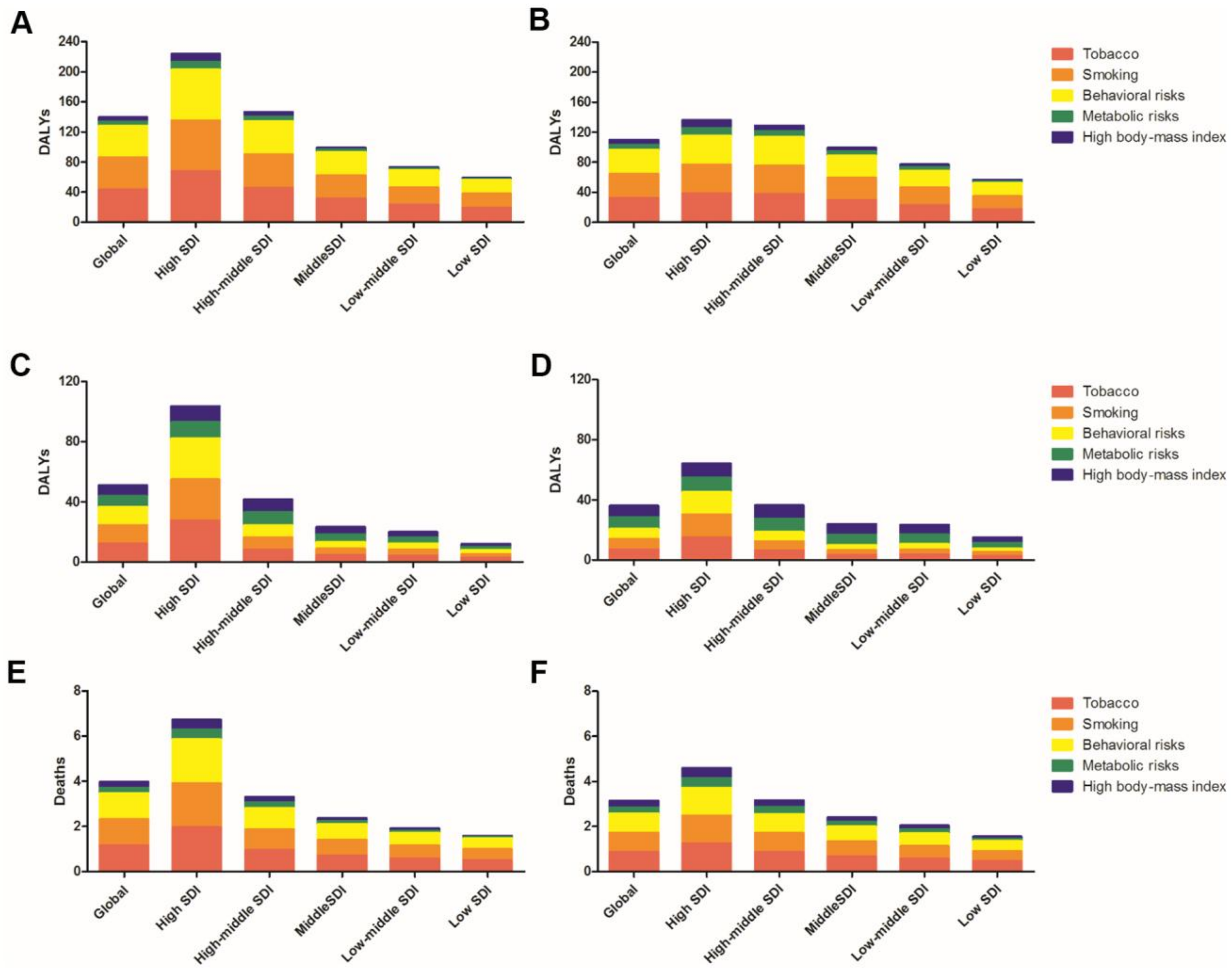

Supplementary Figure 6. The leukemia DALYs attributable to risk factors (**A**, **C**) in males and (**B**, **D**) in females; the leukemia deaths attributable to risk factors (**E**) in males and (**F**) in females.
