# Supplementary material for: Global, regional, and national burdens of leukemia from 1990 to 2017: a systematic analysis of the global burden of disease 2017 study
Source: Aging (Albany NY). 2021 Apr 4;13(7):10468–89. doi: 10.18632/aging.202809 (PMC8064161; doi:10.18632/aging.202809)
Supplement: Supplementary Tables 1, 2 and 3 [file aging-13-202809-s002.pdf]

SUPPLEMENTARY TABLES

Supplementary Table 1. List of international classification of diseases and injuries-10th edition (ICD-10) codes.

Cause	ICD-10
Leukemia	C91-C93.7; C93.9-C95.2; C95.7-C95.92; Z80.6; Z85.6
Acute lymphocytic leukemia	C91.0-C91.02
Acute myeloid leukemia	C92.0-C92.02; C92.3-C92.62; C93.0-C93.02; C94.0-C94.02; C94.2-C94.22
Chronic lymphocytic leukemia	C91.1-C91.12
Chronic myeloid leukemia	C92.1-C92.12
Other leukemia	C91.03-C91.05; C91.2-C91.9; C92.13-C92.2 C92.7-C92.9; C93.1-C93.7; C94.03-C94.1; C94.3-C95.2; C95.7-C95.92; Z80.6; Z85.6

Supplementary Table 2. SDI scales.

Covariate	Lower bound	Upper bound
TFU25	0	3
LDI per capita	250 USD (5.52 log USD)	60,000 USD (11.00 log USD)
EDU15+	0 years	17 years

Supplementary Table 3. Lay description and disability weights.

Health state	Lay description	Estimated disability weights(95%UI)
Leukemia, diagnosis and primary therapy	This person has pain, nausea, fatigue, weight loss and high anxiety	0.288 (0.193, 0.399)
Leukemia, controlled phase	This person has a chronic disease that requires medication every day and causes some worry but minimal interference with daily activities	0.049 (0.031, 0.072)
Leukemia, metastatic	This person has severe pain, extreme fatigue, weight loss and high anxiety	0.451 (0.307, 0.600)
Leukemia, terminal phase with medication	This person has lost a lot of weight and regularly uses strong medication to avoid constant pain. The person has no appetite, feels nauseous, and needs to spend most of the day in bed.	0.540 (0.377, 0.687)
