# Supplementary material for: Global, regional, and national burdens of leukemia from 1990 to 2017: a systematic analysis of the global burden of disease 2017 study
Source: Aging (Albany NY). 2021 Apr 4;13(7):10468–89. doi: 10.18632/aging.202809 (PMC8064161; doi:10.18632/aging.202809)
Supplement: Supplementary Table 4 [file aging-13-202809-s003.doc]

Supplementary Table 4.︱ The EAPCs of leukemia in 195 countries and territories from 1990 to 2017

|  | ASIR | |  | | ASPR | |  | | ASDR | |  | | DALY | |
| --- | --- | --- | --- | --- | --- | --- | --- | --- | --- | --- | --- | --- | --- | --- |
|  | EAPC (95%CI) | P | | EAPC (95%CI) | | P | | EAPC (95%CI) | | P | | EAPC (95%CI) | | P |
| Afghanistan | 0.28 (0.09,0.46) | 0.05 | | 0.33 (0.12,0.54) | | <0.01 | | 0.25 (0.07,0.43) | | <0.01 | | 0.23 (0.00,0.46) | | 0.050 |
| Albania | 0.54 (0.41,0.67) | <0.01 | | 2.51 (2.32,2.59) | | <0.01 | | -0.12 (-0.26,-0.02) | | 0.102 | | -0.40 (-0.54,-0.27) | | <0.01 |
| Algeria | 0.10 (-0.02,0.22) | 0.092 | | 0.42 (0.32,0.51) | | <0.01 | | -0.05 (-0.18,0.09) | | 0.466 | | -0.32 (-0.47,-0.17) | | <0.01 |
| American Samoa | -0.26 (-0.31,-0.20) | <0.01 | | 0.36 (0.27,0.44) | | <0.01 | | -0.61 (-0.69,-0.53) | | <0.01 | | -0.63 (-0.69,-0.56) | | <0.01 |
| Andorra | -0.57 (-0.65,-0.49) | <0.01 | | -0.25 (-0.47,-0.04) | | <0.05 | | -0.58 (-0.62,-0.53) | | <0.01 | | -0.92 (-0.98,-0.86) | | <0.01 |
| Angola | -0.64 (-0.79,-0.49) | <0.01 | | -1.07 (-1.28,-0.86) | | <0.01 | | -0.51 (-0.64,-0.39) | | <0.01 | | -0.90 (-1.08,-0.71) | | <0.01 |
| Antigua and Barbuda | -0.11 (-0.16,-0.07) | <0.01 | | 0.71 (0.64,0.77) | | <0.01 | | -0.61 (0.69,-0.3) | | <0.01 | | -0.84 (-0.53,-0.43) | | <0.01 |
| Argentina | -0.80 (-0.89,-0.71) | <0.01 | | -0.39 (-0.49,-0.29) | | <0.01 | | -0.85 (-0.96,-0.75) | | <0.01 | | -1.03 (-1.11,-0.96) | | <0.01 |
| Armenia | -0.70 (-0.81,-059) | <0.01 | | 0.18 (-0.03,0.38) | | 0.088 | | -0.78 (-0.88,-0.68) | | <0.01 | | -1.49 (-1.60,-1.38) | | <0.01 |
| Australia | -1.79 (-2.08,-1.50) | <0.01 | | -1.07 (-1.51,-0.64) | | <0.01 | | -2.35 (-2.61,-2.08) | | <0.01 | | -2.46 (-2.71,-2.20) | | <0.01 |
| Austria | 1.01 (0.90,1.17) | <0.01 | | 1.18 (0.97,1.39) | | <0.01 | | 0.31 (0.11,0.51) | | <0.01 | | -0.38 (-0.55,-0.21) | | <0.01 |
| Azerbaijan | 0.95 (0.68,1.22) | <0.01 | | 1.66 (1.47,1.84) | | <0.01 | | 0.22 (-0.13,0.57) | | 0.216 | | 0.29 (-0.17,0.75) | | 0.202 |
| Bahrain | -2.60 (-3.04,-2.16) | <0.01 | | -1.29 (-1.63,-0.95) | | <0.01 | | -3.01 (-3.49,-2.53) | | <0.01 | | -2.99 (-3.43,-2.55) | | <0.01 |
| Bangladesh | -1.51 (-1.63,-1.39) | <0.01 | | -1.69 (-1.80,-1.59) | | <0.01 | | -1.58 (-1.69,-1.48) | | <0.01 | | -1.47 (-1.59,-1.35) | | <0.01 |
| Barbados | -0.24 (-0.30,-0.17) | <0.01 | | 0.07 (-0.03,0.17) | | 0.197 | | -0.46 (-0.52,-0.40) | | <0.01 | | -0.52 (-0.59,-0.45) | | <0.01 |
| Belarus | -0.19 (-0.39,-0.02) | 0.075 | | 0.66 (0.29,1.02) | | <0.01 | | -0.76 (-1.04,-0.48) | | <0.01 | | -1.76 (-2.00,-1.53) | | <0.01 |
| Belgium | -0.47 (-0.68,-0.26) | <0.01 | | 0.01 (-0.28,0.29) | | 0.972 | | -0.82 (-1.03,-0.60) | | <0.01 | | -1.09 (-1.41,-0.77) | | <0.01 |
| Belize | -0.25 (-0.49,-0.07) | <0.05 | | -0.32 (-0.51,-0.14) | | <0.01 | | -0.25 (-0.51,0.01) | | 0.062 | | -0.46 (-0.67,-0.25) | | <0.01 |
| Benin | 0.04 (-0.02,0.10) | 0.187 | | -0.04 (-0.11,0.04) | | 0.343 | | 0.06 (0.01,0.10) | | <0.05 | | -0.01 (-0.11,0.09) | | 0.829 |
| Bermuda | -1.28 (-1.42,-1.16) | <0.01 | | 0.83 (0.64,1.02) | | <0.01 | | -2.02 (-2.14,-1.89) | | <0.01 | | -2.35 (-2.56,-2.15) | | <0.01 |
| Bhutan | -0.90 (-1.01,-0.79) | <0.01 | | -1.50 (-1.65,-1.36) | | <0.01 | | -0.76 (-0.86,-0.66) | | <0.01 | | -1.30 (-1.43,-1.16) | | <0.01 |
| Bolivia | -0.54 (-0.74,-0.34) | <0.01 | | -1.03 (-1.21,-0.84) | | <0.01 | | -0.44 (-0.62,-0.26) | | <0.01 | | -0.86 (-1.09,-0.64) | | <0.01 |
| Bosnia and Herzegovina | 0.57 (0.46,0.67) | <0.01 | | 1.93 (1.84,2.03) | | <0.01 | | 0.41 (0.28,0.54) | | <0.01 | | -0.20 (-0.32,-0.07) | | <0.01 |
| Botswana | -0.27 (-0.48,-0.06) | <0.05 | | -0.26 (0.47,-0.05) | | <0.05 | | -0.33 (-0.54,-0.12) | | <0.01 | | -0.58 (-0.77,-0.39) | | <0.01 |
| Brazil | -0.59 (-0.68,-0.51) | <0.01 | | -0.30 (-0.38,-0.23) | | <0.01 | | -0.63 (-0.71,-0.55) | | <0.01 | | -1.01 (-1.11,-0.91) | | <0.01 |
| Brunei | 1.06 (0.81,1.31) | <0.01 | | 1.99 (1.75,2.25) | | <0.01 | | 0.50 (.26,0.75) | | <0.01 | | -0.65 (-0.40,-0.91) | | <0.01 |
| Bulgaria | 1.08 (0.81,1.36) | <0.01 | | 2.23 (1.91,2.55) | | <0.01 | | 0.39 (0.22,0.57) | | <0.01 | | -0.33 (-0.50,-0.16) | | <0.01 |
| Burkina Faso | -0.27 (-0.45,-0.10) | <0.05 | | -0.22 (-0.42,-0.03) | | <0.05 | | -0.30 (-0.46,-0.14) | | <0.01 | | -0.29 (-0.48,-0.10) | | <0.01 |
| Burundi | -0.57 (-0.64,-0.51) | <0.01 | | -0.67 (-0.75,-0.60) | | <0.01 | | -0.49 (-0.55,-0.43) | | <0.01 | | -0.71 (-0.79,-0.63) | | <0.01 |
| Cambodia | -0.49 (-0.52,-0.45) | <0.01 | | -0.77 (-0.81,-0.72) | | <0.01 | | -0.40 (-0.43,-0.37) | | <0.01 | | -0.79 (-0.85,-0.74) | | <0.01 |
| Cameroon | 0.10 (0.00,0.16) | 0.228 | | -0.02 (-0.11,0.07) | | 0.607 | | 0.13 (0.02,0.23) | | <0.05 | | 0.00 (-0.09,0.10) | | 0.947 |
| Canada | -0.87 (-1.04,-0.69) | <0.01 | | -0.25 (-0.47,-0.02) | | <0.05 | | -1.31 (-1.43,-1.18) | | <0.01 | | -1.88 (-2.05,-1.70) | | <0.01 |
| Cape Verde | 0.92 (0.62,1.21) | <0.01 | | 0.80 (0.52,1.07) | | <0.01 | | 0.85 (0.52,1.18) | | <0.01 | | 0.57 (0.29,0.85) | | <0.01 |
| Central African Republic | -0.04 (-0.12,0.05) | 0.383 | | 0.01 (-0.06,0.08) | | 0.842 | | -0.03 (-0.020.05) | | 0.438 | | 0.03 (-0.05,0.12) | | 0.460 |
| Chad | 0.65 (0.54,0.76) | <0.01 | | 0.60 (0.50,0.70) | | <0.01 | | 0.63 (0.52,0.74) | | <0.01 | | 0.68 (0.57,0.79) | | <0.01 |
| Chile | -0.08 (-0.16,0.00) | 0.051 | | 1.16 (1.04,1.29) | | <0.01 | | -0.31 (-0.39,-0.22) | | <0.01 | | -0.83 (-0.91,-0.75) | | <0.01 |
| China | 0.82 (0.62,1.02) | <0.01 | | 2.97 (2.66,3.28) | | <0.01 | | -2.27 (-2.51,-2.04) | | <0.01 | | -2.93 (-3.23,-2.63) | | <0.01 |
| Colombia | -1.11 (-1.27,-0.95) | <0.01 | | -0.53 (-0.81,-0.26) | | <0.01 | | -1.42 (-1.59,-1.25) | | <0.01 | | -1.39 (-1.59,-1.18) | | <0.01 |
| Comoros | -0.10 (-0.21,-0.02) | 0.086 | | -0.30 (-0.43,-0.17) | | <0.01 | | -0.05 (-0.15,0.04) | | 0.261 | | -0.31 (-0.44,-0.17) | | <0.01 |
| Congo | -0.32 (-0.49,-0.16) | <0.01 | | -0.70 (-0.90,-0.50) | | <0.01 | | -0.20 (-0.34,-0.05) | | <0.01 | | -0.56 (-0.77,-0.35) | | <0.01 |
| Costa Rica | -0.05 (-0.22,0.12) | 0.554 | | 0.73 (0.54,0.92) | | <0.01 | | -0.16 (-0.34,0.01) | | 0.067 | | -0.55 (-0.70,-0.40) | | <0.01 |
| Cote d'Ivoire | 0.16 (0.08,0.27) | <0.01 | | 0.10 (-0.01,0.21) | | 0.074 | | 0.20 (0.11,0.29) | | <0.01 | | 0.21 (0.09,0.33) | | <0.01 |
| Croatia | -0.33 (-0.48,-0.19) | <0.01 | | 0.41 (0.26,0.5) | | <0.01 | | -0.58 (-0.75,-0.41) | | <0.01 | | -1.26 (-1.41,-1.11) | | <0.01 |
| Cuba | -1.23 (-1.41,-1.06) | <0.01 | | -0.17 (-0.36,0.01) | | 0.069 | | -1.40 (-1.57,-1.23) | | <0.01 | | -1.79 (1.96,-1.63) | | <0.01 |
| Cyprus | 0.82 (0.60,1.03) | <0.01 | | 2.31 (1.97,2.66) | | <0.01 | | -0.34 (-0.56,-0.14) | | <0.01 | | -0.88 (-1.09,-0.67) | | <0.01 |
| Czech Republic | -0.93 (-1.13,-0.73) | <0.01 | | 0.05 (-0.29,0.39) | | 0.758 | | -1.22 (-1.38,-1.05) | | <0.01 | | -1.96 (-2.10,-1.81) | | <0.01 |
| Democratic Republic of the Congo | -0.12 (-0.19,-0.06) | <0.01 | | -0.23 (-0.29,-0.18) | | <0.01 | | -0.06 (-0.11,-0.010 | | <0.05 | | -0.22 (-0.30,-0.14) | | <0.01 |
| Denmark | -1.44 (-1.84,-1.03) | <0.01 | | -0.80 (-1.16,-0.44) | | <0.01 | | -2.00 (-2.39,-1.60) | | <0.01 | | -2.45 (-2.82,-2.08) | | <0.01 |
| Djibouti | -0.04 (-0.19,0.11) | 0.586 | | -0.45 (-0.66,-0.24) | | <0.01 | | 0.05 (-0.07,0.17) | | 0.419 | | -0.25 (-0.46,-0.05) | | <0.05 |
| Dominica | 0.60 (0.48,0.72) | <0.01 | | 1.07 (0.94,1.21) | | <0.01 | | 0.27 (0.14,0.39) | | <0.01 | | 0.65 (0.50,0.79) | | <0.01 |
| Dominican Republic | 0.15 (-0.01,0.31) | 0.053 | | -0.23 (-0.43,-0.03) | | <0.05 | | 0.10 (-0.03,0.23) | | 0.114 | | -0.31 (-0.51,-0.12) | | <0.01 |
| Ecuador | 1.30 (1.06,1.53) | <0.01 | | 1.18 (0.98,1.39) | | <0.01 | | 1.05 (0.82,1.28) | | <0.01 | | 0.96 (0.74,1.18) | | <0.01 |
| Egypt | -0.07 (-0.16,0.02) | 0.104 | | 0.04 (-0.05,0.12) | | 0.378 | | -0.18 (-0.27,-0.10) | | <0.01 | | -0.38 (-0.49,-0.27) | | <0.01 |
| El Salvador | -0.17 (-0.27,-0.06) | <0.01 | | -0.58 (-0.69,-0.47) | | <0.01 | | -0.11 (-0.22,0.01) | | 0.054 | | -0.65 (-0.77,-0.53) | | <0.01 |
| Equatorial Guinea | -1.53 (-1.76,-1.30) | <0.01 | | -2.09 (-2.38,-1.80) | | <0.01 | | -1.41 (-1.61,-1.22) | | <0.01 | | -2.01 (-2.28,-1.74) | | <0.01 |
| Eritrea | 0.17 (-0.12,0.46) | 0.249 | | 0.06 (-0.16,0.29) | | 0.560 | | 0.19 (-0.10,0.48) | | 0.200 | | 0.12 (-0.21,0.45) | | 0.467 |
| Estonia | 0.99 (0.76,1.22) | <0.01 | | 2.97 (2.60,3.33) | | <0.01 | | -0.34 (-0.57,-0.10) | | <0.01 | | -1.07 (-1.26,-0.88) | | <0.01 |
| Ethiopia | -0.73 (-0.80,-0.66) | <0.01 | | -0.63 (-0.71,-0.55) | | <0.01 | | -0.68 (-0.74,-0.62) | | <0.01 | | -0.89 (-0.97,--0.81) | | <0.01 |
| Federated States of Micronesia | -0.53 (-0.58,-0.48) | <0.01 | | -0.53 (-0.59,-0.47) | | <0.01 | | -0.57 (-0.61,-0.52) | | <0.01 | | -0.62 (-0.68,-0.56) | | <0.01 |
| Fiji | -0.31 (-0.40,-0.20) | <0.01 | | -0.62,-0.73,-0.50 | | <0.01 | | -0.40 (-0.49,-0.30) | | <0.01 | | -0.41 (-0.52,-0.30) | | <0.01 |
| Finland | -0.91 (-1.16,-0.66) | <0.01 | | 0.24 (-0.14,0.63) | | 0.209 | | -2.73 (-2.98,-2.48) | | <0.01 | | -2.67 (-2.90,-2.44) | | <0.01 |
| France | -0.70 (-0.86,-0.55) | <0.01 | | 0.10 (-0.08,0.27) | | 0.282 | | -1.14 (-1.33,-0.94) | | <0.01 | | -1.70 (-1.89,-1.50) | | <0.01 |
| Gabon | 0.15 (0.01,0.30) | <0.05 | | -0.06 (-0.20,0.08) | | 0.382 | | 0.14 (-0.02,.29) | | 0.070 | | 0.10 (-0.05,0.26) | | 0.192 |
| Georgia | 1.26 (0.86,1.66) | <0.01 | | 0.96 (0.52,1.41) | | <0.01 | | 1.73 (1.28,2.18) | | <0.01 | | 0.75 (0.37,1.14) | | <0.01 |
| Germany | -1.75 (-1.88,-1.56) | <0.01 | | -1.48 (-1.66,-1.29) | | <0.01 | | -0.93 (-1.06,-0.80) | | <0.01 | | -1.59 (-1.76,-1.42) | | <0.01 |
| Ghana | -1.70 (-2.07,-1.33) | <0.01 | | -2.14 (-2.59,-1.66) | | <0.01 | | -1.35 (-1.63,-1.07) | | <0.01 | | -2.29 (2.77,-1.80) | | <0.01 |
| Greece | 0.06 (-0.24,0.37) | 0.675 | | 0.59 (0.32,0.86) | | <0.01 | | -0.13 (-0.49,0.22) | | 0.447 | | -0.53 (-0.71,-0.36) | | <0.01 |
| Greenland | -1.34 (-1.41,-1.27) | <0.01 | | -0.61 (-0.73,-0.50) | | <0.01 | | -1.31 (-1.37,-1.24) | | <0.01 | | -1.81 (-1.86,-1.76) | | <0.01 |
| Grenada | -0.47 (-0.75,-0.19) | <0.01 | | 0.07 (-0.24,0.37) | | 0.657 | | -0.54 (-0.85,-0.23) | | <0.01 | | -0.60 (-0.87,-0.33) | | <0.01 |
| Guam | 0.26 (0.09,0.43) | <0.01 | | 0.74 (0.36,1.13) | | <0.01 | | -0.34 (-0.48,-0.19) | | <0.01 | | -0.13 (-0.28,0.020 | | 0.093 |
| Guatemala | 0.66 (0.21,1.10) | <0.01 | | 0.44 (-0.05,0.92) | | 0.075 | | 0.48 (0.01,0.96) | | 0.053 | | 0.55 (0.12,0.98) | | <0.05 |
| Guinea | -0.01 (-0.10,0.09) | 0.906 | | -0.28 (-0.38,-0.17) | | <0.01 | | 0.09 (0.00,0.17) | | <0.05 | | -0.18 (-0.30,-0.07) | | <0.01 |
| Guinea-Bissau | 0.13 (0.06,0.20) | <0.01 | | 0.08 (-0.01,0.17) | | 0.083 | | 0.15 (0.10,0.21) | | <0.01 | | 0.17 (0.07,0.27) | | <0.01 |
| Guyana | 0.12 (-0.07,0.31) | 0.196 | | 0.34 (0.16,0.53) | | <0.01 | | 0.05 (-0.14,0.25) | | 0.590 | | 0.09 (-0.08,0.26) | | 0.294 |
| Haiti | -0.66 (-0.69,-0.63) | <0.01 | | -0.94 (-1.02,-0.85) | | <0.01 | | -0.47 (-0.50,-0.45) | | <0.01 | | -1.00 (-1.04,-0.96) | | <0.01 |
| Honduras | 0.23 (0.09,0.38) | <0.01 | | -0.17 (-0.28,-0.06) | | <0.01 | | 0.12 (0.01,0.24) | | <0.05 | | -0.47 (-0.59,-0.35) | | <0.01 |
| Hungary | -0.37 (-0.87,0.12) | 0.134 | | 0.83 (0.30,1.37) | | <0.01 | | -0.53 (-1.01,-0.05) | | <0.05 | | -1.09 (-1.48,-0.69) | | <0.01 |
| Iceland | -0.96 (-1.08,-0.84) | <0.01 | | -0.47 (-0.1,-0.22) | | <0.01 | | -1.17 (-1.28,-1.06) | | <0.01 | | -1.45 (-1.55,-1.36) | | <0.01 |
| India | -0.40 (-0.50,-0.31) | <0.01 | | -0.85 (-0.94,-0.75) | | <0.01 | | -0.25 (-0.33,-0.16) | | <0.01 | | -0.88 (-1.00,-0.75) | | <0.01 |
| Indonesia | 0.07 (-0.04,0.19) | 0.207 | | -0.38 (-0.47,-0.28) | | <0.01 | | 0.24 (0.10,0.37) | | <0.01 | | -0.40 (-0.50,-0.29) | | <0.01 |
| Iran | 0.98 (0.62,1.34) | <0.01 | | 2.43 (2.01,2.86) | | <0.01 | | 0.45 (0.09,0.81) | | <0.05 | | 0.20 (-0.18,0.58) | | 0.297 |
| Iraq | -1.92 (-2.22,-1.62) | <0.01 | | -1.67 (-1.97,-1.37) | | <0.01 | | -2.12 (-2.43,-1.81) | | <0.01 | | -1.98 (-2.32,-1.63) | | <0.01 |
| Ireland | -0.18 (-0.71,0.35) | 0.494 | | 0.99 (0.26,1.73) | | <0.01 | | -1.09 (-1.61,-0.57) | | <0.01 | | -1.50 (-2.11,-0.88) | | <0.01 |
| Israel | -0.92 (-1.23,-0.62) | <0.01 | | 0.09 (-0.33,0.51) | | 0.669 | | -1.17 (-1.48,-0.86) | | <0.01 | | -1.45 (-1.79,-1.11) | | <0.01 |
| Italy | -0.46 (-0.58,-0.34) | <0.01 | | -0.10 (-0.28,0.09) | | 0.302 | | -0.83 (-0.95,-0.71) | | <0.01 | | -1.56 (-1.65,-1.47) | | <0.01 |
| Jamaica | 1.53 (1.02,2.03) | <0.01 | | 1.55 (1.04,2.06) | | <0.01 | | 1.55 (1.04,2.07) | | <0.01 | | 1.31 (0.86,1.77) | | <0.01 |
| Japan | -0.40 (-0.50,-0.31) | <0.01 | | 0.15 (0.02,0.28) | | <0.05 | | -1.20 (-1.25,-1.15) | | <0.01 | | -2.03 (-2.11,-1.94) | | <0.01 |
| Jordan | -0.74 (-0.90,-0.57) | <0.01 | | -0.53 (-0.70,-0.35) | | <0.01 | | -0.83 (-0.99,-0.67) | | <0.01 | | -1.88 (-2.16,-1.60) | | <0.01 |
| Kazakhstan | -0.57 (-0.73,-0.41) | <0.01 | | 0.39 (0.15,0.62) | | <0.01 | | -0.71 (-0.95,-0.46) | | <0.01 | | -1.35 (-1.58,-1.12) | | <0.01 |
| Kenya | 0.89 (0.30,0.48) | <0.01 | | 0.37 (0.29,0.45) | | <0.01 | | 0.36 (0.27,0.46) | | <0.01 | | 0.40 (0.30,0.49) | | <0.01 |
| Kiribati | 0.01 (-0.08,0.10) | 0.795 | | -0.06 (-0.17,0.05) | | 0.295 | | 0.01 (-0.01,0.11) | | 0.814 | | -0.02 (-0.12,0.08) | | 0.649 |
| Kuwait | -1.32 (-1.64,-1.00) | <0.01 | | -0.67 (-0.90,-0.44) | | <0.01 | | -1.77 (-2.13,-1.40) | | <0.01 | | -2.22 (2.58,-1.86) | | <0.01 |
| Kyrgyzstan | -1.42 (-1.57,-1.28) | <0.01 | | -1.99 (-2.15,-1.86) | | <0.01 | | -1.35 (-1.48,-1.23) | | <0.01 | | -1.91 (-2.06,-1.77) | | <0.01 |
| Laos | -0.48 (-0.57,-0.39) | <0.01 | | -0.70 (-0.80,-0.61) | | <0.01 | | -0.43 (-0.52,-0.34) | | <0.01 | | -0.75 (-0.86,-0.64) | | <0.01 |
| Latvia | 0.81 (0.59,1.04) | <0.01 | | 2.22 (1.88,2.55) | | <0.01 | | -0.66 (-0.80,-0.52) | | <0.01 | | -1.21 (-1.36,-1.06) | | <0.01 |
| Lebanon | 0.36 (0.10,0.63) | <0.01 | | 1.74 (1.42,2.07) | | <0.01 | | -1.07 (-1.25,-0.90) | | <0.01 | | -1.06 (-1.26,-0.85) | | <0.01 |
| Lesotho | 1.06 (0.66,1.46) | <0.01 | | 0.88 (0.52,1.24) | | <0.01 | | 1.06 (0.68,1.45) | | <0.01 | | 1.08 (0.67,1.50) | | <0.01 |
| Liberia | -0.31 (-0.54,-0.13) | <0.01 | | -0.50 (-0.72,-0.26) | | <0.01 | | -0.14 (-0.30,0.02) | | 0.088 | | -0.50 (-0.77,-0.23) | | <0.01 |
| Libya | 1.04 (0.81,1.28) | <0.01 | | 1.83 (1.55,2.11) | | <0.01 | | 0.37 (0.19,0.54) | | <0.01 | | 0.55 (0.46,0.75) | | <0.01 |
| Lithuania | 0.56 (0.32,0.80) | <0.01 | | 1.33 (1.03,1.63) | | <0.01 | | -0.56 (-0.78,-0.34) | | <0.01 | | -1.07 (-1.33,-0.82) | | <0.01 |
| Luxembourg | -0.75 (-0.86,-0.64) | <0.01 | | -0.46 (-0.71,-0.21) | | <0.01 | | -1.19 (-1.28,-1.12) | | <0.01 | | -1.83 (-1.93,-1.73) | | <0.01 |
| Macedonia | 0.12 (0.02,0.22) | <0.05 | | 1.47 (1.34,1.59) | | <0.01 | | -0.26 (-0.35,-0.16) | | <0.01 | | -0.59 (-0.72,-0.46) | | <0.01 |
| Madagascar | -0.50 (-0.64,-0.36) | <0.01 | | -0.74 (-0.92,-0.55) | | <0.01 | | -0.30 (-0.40,-0.21) | | <0.01 | | -0.97 (-1.21,-0.74) | | <0.01 |
| Malawi | -0.36 (-0.57,-0.15) | <0.01 | | -0.43 (-0.65,-0.20) | | <0.01 | | -0.38 (-0.58,-0.18) | | <0.01 | | -0.32 (-0.55,-0.08) | | <0.05 |
| Malaysia | -0.08 (-0.17,-0.04) | <0.01 | | 0.35 (0.18,0.52) | | <0.01 | | -0.53 (-0.60,-0.45) | | <0.01 | | -1.04 (-1.13,-0.94) | | <0.01 |
| Maldives | -1.05 (-1.16,-0.94) | <0.01 | | -0.17 (-0.36,0.02) | | 0.08 | | -2.06 (-2.23,-1.89) | | <0.01 | | -2.31 (-2.43,-2.20) | | <0.01 |
| Mali | -0.39 (-0.50,-0.29) | <0.01 | | -0.49 (-0.63,0.35) | | <0.01 | | -0.34 (-0.42,-0.26) | | <0.01 | | -0.51 (-0.65,-0.38) | | <0.01 |
| Malta | -0.19 (0.28,-0.09) | <0.01 | | 1.35 (1.20,1.51) | | <0.01 | | -0.72 (-0.87,-0.58) | | <0.01 | | -0.74 (-0.89,-0.59) | | <0.01 |
| Marshall Islands | 0.14 (0.01,0.28) | <0.05 | | 0.17 (0.03,0.30) | | <0.05 | | 0.13 (0.00,0.26) | | 0.050 | | 0.05 (-0.12,0.21) | | 0.580 |
| Mauritania | 0.21 (0.18,0.25) | <0.01 | | 0.12 (0.09,0.15) | | <0.01 | | 0.21 (0.17,0.25) | | <0.01 | | 0.15 (0.13,0.20) | | <0.01 |
| Mauritius | -0.47 (-0.68,-0.26) | <0.01 | | -0.37 (-0.57,-0.16) | | <0.01 | | -1.19 (-1.36,-1.01) | | <0.01 | | -1.35 (-1.50,-1.19) | | <0.01 |
| Mexico | -0.05 (-0.17,0.07) | 0.408 | | 0.52 (0.37,0.67) | | <0.01 | | -0.23 (-0.32,-0.14) | | <0.01 | | -0.33 (-0.45,-0.21) | | <0.01 |
| Moldova | -1.81 (-2.06,-1.56) | <0.01 | | -1.50 (-1.91,-1.09) | | <0.01 | | -1.99 (-2.16,-1.82) | | <0.01 | | -2.70 (-2.87,-2.53) | | <0.01 |
| Mongolia | -0.34 (-0.53,-0.15) | <0.01 | | -0.19 (-0.36,-0.02) | | <0.05 | | -0.47 (-0.66,-0.29) | | <0.01 | | -0.57 (-0.78,-0.37) | | <0.01 |
| Montenegro | -0.50 (-0.61,-0.39) | <0.01 | | 0.86 (0.72,0.99) | | <0.01 | | -0.54 (-0.66,-0.42) | | <0.01 | | -0.95 (-1.13,-0.77) | | <0.01 |
| Morocco | -0.03 (-0.05,0.00) | 0.052 | | 0.15 (0.08,0.21) | | <0.01 | | -0.13 (-0.16,-0.10) | | <0.01 | | -0.27 (-0.31,-0.24) | | <0.01 |
| Mozambique | -0.43 (-0.73,-0.14) | <0.01 | | -0.74 (-1.09,-0.39) | | <0.01 | | -0.43 (-0.69,-0.17) | | <0.01 | | -0.49 (-0.83,-0.150 | | <0.01 |
| Myanmar | -0.64 (-0.81,-0.46) | <0.01 | | -0.90 (-1.08,-0.72) | | <0.01 | | -0.55 (-0.74,-0.36) | | <0.01 | | -0.94 (-1.14,-0.74) | | <0.01 |
| Namibia | -0.82 (-1.21,-0.43) | <0.01 | | -0.89 (-1.22,-0.56) | | <0.01 | | -0.86 (-1.25,-0.47) | | <0.01 | | -0.84 (-1.24,-0.44) | | <0.01 |
| Nepal | -0.46 (-0.62,-0.30) | <0.01 | | -0.94 (-1.14,-0.73) | | <0.01 | | -0.33 (-0.47,-0.18) | | <0.01 | | -0.76 (-0.94,-0.57) | | <0.01 |
| Netherlands | -0.14 (-0.26,-0.01) | 0.297 | | -0.54 (-0.87,-0.20) | | <0.01 | | -0.45 (-0.60,-0.31) | | <0.01 | | -1.32 (-1.51,-1.13) | | <0.01 |
| New Zealand | -0.80 (-0.93,-0.68) | <0.01 | | -0.30 (-0.49,-0.12) | | <0.01 | | -1.02 (-1.11,-0.93) | | <0.01 | | -1.38 (-1.47,-1.28) | | <0.01 |
| Nicaragua | -0.62 (-0.74,-0.50) | <0.01 | | -0.73 (-0.83,0.63) | | <0.01 | | -0.63 (-0.74,-0.52) | | <0.01 | | -1.94 (-1.19,-0.99) | | <0.01 |
| Niger | -0.62 (-0.72,-0.52) | <0.01 | | -0.73 (-0.83,-0.62) | | <0.01 | | -0.47 (-0.56,-0.38) | | <0.01 | | -0.88 (-1.00,-0.75) | | <0.01 |
| Nigeria | -0.04 (-0.14,0.07) | 0.456 | | -0.22 (-0.37,-0.07) | | <0.01 | | -0.02 (-0.11,0.07) | | 0.715 | | -0.19 (-0.33,-0.05) | | <0.05 |
| North Korea | 0.29 (0.18,0.40) | <0.01 | | 0.43 (0.32,0.54) | | <0.01 | | 0.14 (0.01,0.28) | | <0.05 | | 0.10 (-0.04,0.24) | | 0.172 |
| Northern Mariana Islands | 0.22 (0.03,0.41) | <0.05 | | 0.90 (0.46,1.35) | | <0.01 | | -0.69 (-0.80,-0.58) | | <0.01 | | -0.70 (-0.84,-0.55) | | <0.01 |
| Norway | 0.17 (-0.06,0.41) | 0.142 | | 0.08 (-0.24,0.39) | | 0.515 | | -0.82 (-0.97,-0.67) | | <0.01 | | -1.36 (-1.55,-1.18) | | <0.01 |
| Oman | -0.03 (-0.22,0.17) | 0.785 | | 0.75 (0.43,.07) | | <0.01 | | -0.69 (-0.86,-0.53) | | <0.01 | | -0.94 (-1.16,-0.72) | | <0.01 |
| Pakistan | 0.71 (0.57,0.85) | <0.01 | | 0.45 (0.31,0.59) | | <0.01 | | 0.69 (0.57,0.82) | | <0.01 | | 0.67 (0.52,0.81) | | <0.01 |
| Palestine | -0.55 (-0.59,-0.52) | <0.01 | | -0.56 (-0.60,-0.52) | | <0.01 | | -0.59 (-0.63,-0.55) | | <0.01 | | -0.84 (-0.90,-0.78) | | <0.01 |
| Panama | -0.54 (-0.84,-0.23) | <0.01 | | 0.28 (-0.05,0.61) | | 0.095 | | -0.80 (-1.10,-0.50) | | <0.01 | | -0.74 (-1.45,-0.43) | | <0.01 |
| Papua New Guinea | -0.09 (-0.15,-0.03) | <0.01 | | -0.07 (-0.15,0.01) | | 0.073 | | -0.04 (-0.09,0.01) | | 0.098 | | -0.14 (-0.21,-0.06) | | <0.01 |
| Paraguay | -0.15 (-0.37,0.07) | 0.173 | | -0.87 (-1.14,-0.60) | | <0.01 | | -0.09 (-0.28,0.09) | | 0.303 | | -0.62 (-0.88,-0.37) | | <0.01 |
| Peru | -0.22 (-0.35,-0.08) | <0.01 | | -0.17 (-0.36,0.02) | | 0.072 | | -0.45 (-0.59,-0.32) | | <0.01 | | -0.74 (-0.88,-0.61) | | <0.01 |
| Philippines | 1.00 (0.77,1.23) | <0.01 | | 0.71 (0.43,0.99) | | <0.01 | | 0.89 (0.67,1.11) | | <0.01 | | 0.70 (0.44,0.97) | | <0.01 |
| Poland | -0.17 (-0.31,-0.04) | <0.05 | | 1.27 (1.12,1.42) | | <0.01 | | -0.28 (-0.38,-0.18) | | <0.01 | | -1.12 (-1.20,-1.04) | | <0.01 |
| Portugal | -1.21 (-1.41,-1.02) | <0.01 | | 0.01 (-0.18,0.19) | | 0.952 | | -1.27 (-1.43,-1.10) | | <0.01 | | -2.33 (-2.49,-2.18) | | <0.01 |
| Puerto Rico | -0.69 (-0.80.-0.59) | <0.01 | | 0.50 (0.34,0.67) | | <0.01 | | -1.10 (-1.20,-1.00) | | <0.01 | | -1.39 (-1.50,-1.28) | | <0.01 |
| Qatar | -1.38 (-1.66,-1.10) | <0.01 | | 0.66 (0.49,0.83) | | <0.01 | | -2.17 (-2.51,-1.83) | | <0.01 | | -2.00 (-2.28,-1.71) | | <0.01 |
| Romania | 0.23 (0.12,0.34) | <0.01 | | 1.59 (1.42,1.77) | | <0.01 | | 0.18 (0.09,0.28) | | <0.01 | | -0.94 (-1.04,-0.84) | | <0.01 |
| Russian Federation | 0.38 (0.18,0.59) | <0.01 | | 1.36 (1.03,1.69) | | <0.01 | | -0.17 (-0.42,0.09) | | 0.189 | | -1.03 (-1.27,-0.79) | | <0.01 |
| Rwanda | -0.77 (-0.98,-0.57) | <0.01 | | -0.91 (-1.18,-0.63) | | <0.01 | | -0.65 (-0.81,-0.50) | | <0.01 | | -1.00 (-1.27,-0.73) | | <0.01 |
| Saint Lucia | -1.02 (-1.14,-0.89) | <0.01 | | -0.71 (-0.84,-0.59) | | <0.01 | | -1.14 (-1.29,-1.00) | | <0.01 | | -1.24 (-1.36,-1.12) | | <0.01 |
| Saint Vincent and the Grenadines | -0.21 (-0.36,-0.05) | <0.05 | | -0.08 (-0.24,0.08) | | 0.298 | | -0.30 (-0.46,-0.13) | | <0.01 | | -0.22 (-0.41,-0.04) | | <0.05 |
| Samoa | -0.54 (-0.59,-0.49) | <0.01 | | -0.58 (-0.64,-0.52) | | <0.01 | | -0.56 (-0.61,-0.50) | | <0.01 | | -0.78 (-0.84,-0.72) | | <0.01 |
| Sao Tome and Principe | 0.34 (0.26,0.42) | <0.01 | | 0.14 (0.05,0.240 | | <0.01 | | 0.43 (0.36,0.49) | | <0.01 | | 0.15 (0.05,0.25) | | <0.01 |
| Saudi Arabia | 0.93 (0.82,1.04) | <0.01 | | 1.46 (1.31,1.61) | | <0.01 | | 0.50 (0.31,0.69) | | <0.01 | | -0.22 (-0.36,-0.09) | | <0.01 |
| Senegal | 0.47 (0.39,0.55) | <0.01 | | 0.35 (0.25,0.44) | | <0.01 | | 0.58 (0.51,0.64) | | <0.01 | | 0.38 (0.27,0.48) | | <0.01 |
| Serbia | 0.01 (-0.16,0.19) | 0.886 | | 0.28 (0.11,0.45) | | <0.01 | | -0.08 (-0.22,0.06) | | 0.254 | | -1.03 (-1.18,0.88) | | <0.01 |
| Seychelles | 0.62 (0.41,0.83) | <0.01 | | 1.23 (1.04,1.43) | | <0.01 | | 0.11 (-0,10,0.32) | | 0.277 | | -0.03 (-0.24,-0.19) | | 0.810 |
| Sierra Leone | 0.13 (0.07,0.20) | <0.01 | | -0.05 (-0.15,0.06) | | 0.375 | | 0.28 (0.23,0.32) | | <0.01 | | -0.06 (-0.18,0.06) | | 0.322 |
| Singapore | -0.59 (-0.72,-0.46) | <0.01 | | 1.79 (1.48,2.10) | | 0.375 | | -1.69 (-1.81,-1.56) | | <0.01 | | -2.09 (-2.20,-1.98) | | <0.01 |
| Slovakia | 1.80 (0.02,2.00) | <0.01 | | 3.60 (3.39,3.81) | | <0.01 | | -0.02 (-0.20,0.16) | | 0.837 | | -0.35 (-0.50,-0.19) | | <0.01 |
| Slovenia | -0.30 (-0.48,-0.10) | <0.01 | | 0.69 (0.40,0.98) | | <0.01 | | -0.32 (-0.50,-0.14) | | <0.01 | | -1.10 (-1.26,-0.93) | | <0.01 |
| Solomon Islands | -0.28 (-0.31,-0.26) | <0.01 | | -0.29 (-0.33,-0.25) | | <0.01 | | -0.25 (-0.28,-0.23) | | <0.01 | | -0.35 (-0.39,-0.310 | | <0.01 |
| Somalia | 0.51 (0.32,0.70) | <0.01 | | 0.19 (-0.06,0.44) | | 0.126 | | 0.60 (0.44,0.75) | | <0.01 | | 0.47 (0.23,0.71) | | <0.01 |
| South Africa | -0.64 (-1.03,-0.24) | <0.01 | | -0.86 (-1.24,-0.48) | | <0.01 | | -0.53 (-0.89,-0.16) | | <0.01 | | -0.99 (-1.48,-0.49) | | <0.01 |
| South Korea | -0.28 (-0.45,-0.11) | <0.01 | | 1.66 (1.40,1.92) | | <0.01 | | -1.67 (-1.82,-1.53) | | <0.01 | | -2.78 (-2.90,-2.67) | | <0.01 |
| South Sudan | 0.44 (0.25,0.64) | <0.01 | | 0.47 (0.22,0.71) | | <0.01 | | 0.36 (0.19,0.52) | | <0.01 | | 0.58 (0.32,0.83) | | <0.01 |
| Spain | -0.26 (-0.31,-0.21) | <0.01 | | 0.70 (0.49,0.92) | | <0.01 | | -1.04 (-1.17,-0.92) | | <0.01 | | -1.84 (-1.94,-1.75) | | <0.01 |
| Sri Lanka | 0.09 (-0.02,0.19) | 0.116 | | 0.69 (0.53,0.85) | | <0.01 | | -0.72 (-0.85,-0.60) | | <0.01 | | -0.84 (-0.96,-0.72) | | <0.01 |
| Sudan | -0.05 (-0.14,0.05) | 0.307 | | -0.13 (-0.26,0.01) | | 0.530 | | -0.03 (-0.11,0.06) | | 0.516 | | -0.19 (-0.30,-0.07) | | <0.01 |
| Suriname | -0.47 (-0.56,-0.38) | <0.01 | | -0.56 (-0.65,-0.47) | | <0.01 | | -0.41 (-0.51,-0.31) | | <0.01 | | -0.75 (-0.84,-0.65) | | <0.01 |
| Swaziland | 0.29 (-0.08,0.67) | 0.123 | | 0.06 (-0.23,0.35) | | 0.698 | | 0.31 (-0.06,0.69) | | 0.100 | | 0.44 (0.00,0.88) | | <0.05 |
| Sweden | 0.24 (0.11,0.37) | <0.01 | | 0.38 (0.15,0.62) | | <0.01 | | -0.32 (-0.41,-0.23) | | <0.01 | | -0.79 (-0.89,-0.68) | | <0.01 |
| Switzerland | -0.85 (-1.17,-0.53) | <0.01 | | -0.86 (-1.25,-0.47) | | <0.01 | | -0.91 (-1.18,-0.63) | | <0.01 | | -1.56 (-1.84,-1.28) | | <0.01 |
| Syria | -1.26 (-1.56,-0.95) | <0.01 | | -0.99 (-1.29,-0.69) | | <0.01 | | -1.36 (-1.65,-1.07) | | <0.01 | | -1.75 (-2.09,-1.40) | | <0.01 |
| Taiwan (Province of China) | 1.39 (1.24,1.52) | <0.01 | | 3.00 (2.75,3.25) | | <0.01 | | 0.16 (0.05,0.27) | | <0.01 | | -0.50 (-0.64,-0.35) | | <0.01 |
| Tajikistan | -0.71 (-1.00,-0.42) | <0.01 | | -0.95 (-1.27,-0.64) | | <0.01 | | -0.68 (-0.96,-0.41) | | <0.01 | | -0.95 (1.23,-0.67) | | <0.01 |
| Tanzania | 0.40 (0.31,0.50) | <0.01 | | 0.46 (0.36,0.56) | | <0.01 | | 0.28 (0.19,0.37) | | <0.01 | | 0.50 (0.39,0.60) | | <0.01 |
| Thailand | -0.35 (-0.53,-0.16) | <0.01 | | 0.16 (-0.02,0.34) | | 0.089 | | -1.15 (-1.34,-0.96) | | <0.01 | | -1.45 (-1.73,-1.18) | | <0.01 |
| The Bahamas | -0.28 (-0.40,-0.16) | <0.01 | | 0.26 (0.16,0.37) | | <0.01 | | -0.41 (-0.53,-0.29) | | <0.01 | | -0.51 (-0.65,-0.38) | | <0.01 |
| The Gambia | 0.51 (0.44,0.57) | <0.01 | | 0.35 (0.28,0.41) | | <0.01 | | 0.58 (0.51,0.65) | | <0.01 | | 0.48 (0.39,0.56) | | <0.01 |
| Timor-Leste | 0.28 (0.18,0.37) | <0.01 | | -0.21 (-0.33,-0.08) | | <0.01 | | 0.38 (0.32,0.44) | | <0.01 | | -0.19 (-0.30,-0.07) | | <0.01 |
| Togo | -0.22 (-0.33,-0.12) | <0.01 | | -0.33 (-0.41,-0.25) | | <0.01 | | -0.13 (-0.23,-0.02) | | <0.05 | | -0.35 (-0.45,-0.26) | | <0.01 |
| Tonga | 0.10 (0.02,0.18) | 0.201 | | 0.19 (0.13,0.24) | | <0.01 | | -0.07 (-0.15,0.02) | | 0.131 | | 0.22 (0.10,0.34) | | <0.01 |
| Trinidad and Tobago | -1.02 (-1.16,-0.88) | <0.01 | | -0.36 (-0.50,-0.21) | | <0.01 | | -1.26 (-1.40,-1.11) | | <0.01 | | -1.25 (-1.38,-1.12) | | <0.01 |
| Tunisia | -0.54 (-0.61,-0.47) | <0.01 | | -0.12 (-0.17,-0.08) | | <0.01 | | -0.86 (-0.95,-0.77) | | <0.01 | | -1.21 (-1.28,-1.15) | | <0.01 |
| Turkey | -1.00 (-1.12,-0.88) | <0.01 | | 0.53 (0.33,0.72) | | <0.01 | | -1.47 (1.61,-1.33) | | <0.01 | | -1.92 (-2.00,-1.83) | | <0.01 |
| Turkmenistan | -0.22 (-0.44,-0.01) | <0.05 | | -0.36 (-0.68,-0.03) | | <0.05 | | -0.26 (-0.40,-0.11) | | <0.01 | | -0.01 (-1.05,-0.55) | | <0.01 |
| Uganda | 0.24 (0.13,0.36) | <0.01 | | 0.16 (0.03,0.29) | | <0.05 | | 0.28 (0.18,0.38) | | <0.01 | | 0.21 (0.07,0.36) | | <0.01 |
| Ukraine | -0.56 (-0.94,-0.17) | <0.01 | | 0.42 (-0.10,0.94) | | 0.112 | | -1.26 (-1.59,0.93) | | <0.01 | | -2.05 (-2.51,-1.58) | | <0.01 |
| United Arab Emirates | 0.26 (0.16,0.36) | <0.01 | | 1.13 (1.01,1.25) | | <0.01 | | -0.06 (-0.17,0.05) | | 0.262 | | -0.04 (-0.13,-0.04) | | 0.285 |
| United Kingdom | -0.78 (-0.94,-0.61) | <0.01 | | -0.72 (-0.94,-0.50) | | <0.01 | | -1.11 (-1.22,-1.00) | | <0.01 | | -1.73 (-1.85,-1.61) | | <0.01 |
| United States | -0.59 (-0.78,-0.40) | <0.01 | | -0.44 (-0.69,-0.19) | | <0.01 | | -0.95 (-1.04,-0.85) | | <0.01 | | -1.49 (-1.58,-1.40) | | <0.01 |
| Uruguay | -0.70 (-0.81,-0.59) | <0.01 | | -0.14 (-0.29,0.02) | | 0.078 | | -0.70 (-0.81,-0.59) | | <0.01 | | -1.15 (-1.27,-1.03) | | <0.01 |
| Uzbekistan | -0.34 (-0.51,-0.17) | <0.01 | | -0.44 (-0.62,-0.27) | | <0.01 | | -0.40 (-0.56,-0.25) | | <0.01 | | -1.03 (-1.26,-0.78) | | <0.01 |
| Vanuatu | 0.31 (0.23,0.39) | <0.01 | | 0.26 (0.17,0.34) | | <0.01 | | 0.37 (0.29,0.46) | | <0.01 | | 0.23 (0.14,0.33) | | <0.01 |
| Venezuela | -0.64 (-0.74,-054) | <0.01 | | 0.08 (-0.10,0.26) | | 0.378 | | -0.92 (-1.02,-0.83) | | <0.01 | | -1.03 (-1.14,-0.91) | | <0.01 |
| Vietnam | 0.57 (0.44,0.70) | <0.01 | | 0.65 (0.49,0.81) | | <0.01 | | 0.27 (0.15,0.40) | | <0.01 | | 0.23 (0.10,0.37) | | <0.01 |
| Virgin Islands, U.S. | 0.29 (0.15,0.42) | <0.01 | | 1.21 (1.07,1.35) | | <0.01 | | -0.18 (-0.34,-0.03) | | <0.01 | | -0.37 (-0.55,-0.19) | | <0.01 |
| Yemen | 0.28 (0.20,0.37) | <0.01 | | 0.23 (0.15,0.31) | | <0.01 | | 0.31 (0.23,0.39) | | <0.01 | | 0.10 (0.01,0.18) | | <0.05 |
| Zambia | -0.67 (-0.79,-0.55) | <0.01 | | -0.74 (-0.87,-0.60) | | <0.01 | | -0.67 (-0.79,-0.55) | | <0.01 | | -0.72 (-0.85,-0.59) | | <0.01 |
| Zimbabwe | 0.96 (0.46,1.47) | <0.01 | | 1.28 (0.84,.73) | | <0.01 | | 0.89 (0.40,1.38) | | <0.01 | | 1.28 (0.72,1.84) | | <0.01 |
